# Kinesin-5 Eg5 is essential for spindle assembly, chromosome stability and organogenesis in development

**DOI:** 10.1038/s41420-022-01281-1

**Published:** 2022-12-13

**Authors:** Wen-Xin Yu, Yu-Kun Li, Meng-Fei Xu, Chen-Jie Xu, Jie Chen, Ya-Lan Wei, Zhen-Yu She

**Affiliations:** 1 Department of Cell Biology and Genetics, The School of Basic Medical Sciences, Fujian Medical University, 350122 Fuzhou, Fujian China; 2 Key Laboratory of Stem Cell Engineering and Regenerative Medicine, Fujian Province University, 350122 Fuzhou, Fujian China; 3 Medical Research Center, Fujian Maternity and Child Health Hospital, 350001 Fuzhou, Fujian China; 4 College of Clinical Medicine for Obstetrics & Gynecology and Pediatrics, Fujian Medical University, 350122 Fuzhou, Fujian China

**Keywords:** Mitosis, Immunoproliferative disorders

## Abstract

Chromosome stability relies on bipolar spindle assembly and faithful chromosome segregation during cell division. Kinesin-5 Eg5 is a plus-end-directed kinesin motor protein, which is essential for spindle pole separation and chromosome alignment in mitosis. Heterozygous *Eg5* mutations cause autosomal-dominant microcephaly, primary lymphedema, and chorioretinal dysplasia syndrome in humans. However, the developmental roles and cellular mechanisms of Eg5 in organogenesis remain largely unknown. In this study, we have shown that Eg5 inhibition leads to the formation of the monopolar spindle, chromosome misalignment, polyploidy, and subsequent apoptosis. Strikingly, long-term inhibition of Eg5 stimulates the immune responses and the accumulation of lymphocytes in the mouse spleen through the innate and specific immunity pathways. Eg5 inhibition results in metaphase arrest and cell growth inhibition, and suppresses the formation of somite and retinal development in zebrafish embryos. Our data have revealed the essential roles of kinesin-5 Eg5 involved in cell proliferation, chromosome stability, and organogenesis during development. Our findings shed a light on the cellular basis and pathogenesis in microcephaly, primary lymphedema, and chorioretinal dysplasia syndrome of *Eg5*-mutation-positive patients.

## Introduction

During cell division, genetic information is equally distributed into daughter cells by the mitotic spindle and microtubule-associated proteins. Kinesin-5 family motor protein Eg5 (also refers to as KIF11) is a homotetrameric, slow processive, plus-end-directed motor protein required for bipolar spindle assembly and chromosome alignment during mitosis [1–3]. Kinesin-5 Eg5 motors are evolutionarily conserved in eukaryotes, which consist of an N-terminal motor domain, a central stalk domain, and a C-terminal tail domain [4, 5]. Eg5 forms a bipolar homotetramer [1, 2, 6–9], crosslinks and slides antiparallel microtubules, and then generates an outward pushing force to separate spindle poles [10, 11]. Kinesin-5 motors are required for the maintenance of the bipolar spindle in fungi, *Xenopus*, *Drosophila*, and humans [2, 12, 13]. Chemical inhibition or genetic deletion of kinesin-5 motors results in the collapse of the bipolar spindle into the monopolar spindle [2, 5, 13].

Eg5 is essential for the establishment of spindle bipolarity and the separation of the spindle poles [10, 11]. During early mitosis, Eg5 crosslinks the microtubules and separates the spindle poles through its motor activity [14]. Eg5 inhibition by specific antibodies in embryos results in the collapse of mitotic spindles and the formation of mono-asters [2, 15]. Eg5 ablation leads to the activation of the spindle assembly checkpoint, the mitotic arrest at the G_2_/M phase, and subsequent cell death [16]. Kinesin-5 and dynein function together to assemble the spindle into the proper size and shape [17]. The inhibition of kinesin-5 and dynein lead to a less stiff and more mechanically homogenous spindle [17, 18].

In humans, heterozygous *Eg5* mutations result in syndromic autosomal-dominant MLCRD (microcephaly, primary lymphedema, and chorioretinal dysplasia) syndrome (MIM No.152950) [19]. The identifications of *Eg5* mutations in patients suggest that the *Eg5* gene is essential for the development and maintenance of the central nervous system, and the retinal and lymphatic structures [19–21]. Accumulating studies have shown the existence of a link between Eg5 and the development of multiple organs [20–22]; however, the molecular and cellular basis for how the established mitotic functions of Eg5 can account for different phenotypes of MLCRD syndrome during organogenesis and development remains obscure.

In this study, we have revealed that the evolutionarily conserved *Eg5* gene is widely expressed in multiple organs. We have constructed the Eg5-inhibition mouse and zebrafish models and found that Eg5 inhibition results in the collapse of the bipolar spindle, chromosome misalignment, and the activation of the spindle assembly checkpoint. Eg5 inhibition leads to the formation of polyploid and apoptotic cells, which usually occurs in the proliferating cells in multiple tissues. Interestingly, we have found that Eg5 inhibition leads to the increase of lymphocytes in both the spleen and the blood, suggesting the immune responses and the activation of the innate and adaptive immunity pathways. Our findings have also illustrated that Eg5 is responsible for the somite and retinal development during organogenesis. Our data have provided the cellular mechanisms underlying the activation of immune responses after Eg5 inhibition, which is helpful to clarify the pathogenesis in microcephaly, primary lymphedema, and chorioretinal dysplasia syndrome of the patients with *Eg5* mutations.

## Results

### Eg5 proteins were located at spindle microtubules and were required for the formation of bipolar spindle

During prophase, Eg5 proteins were mainly enriched at the centrosomes in HeLa cells (Fig. S1a). Eg5 proteins were gradually distributed at the spindle microtubules and also accumulated at the spindle poles during metaphase (Fig. S1a, b). During anaphase, Eg5 proteins were located around the separating chromosomes and distributed at the microtubules during anaphase (Fig. S1a, c). The localization patterns of Eg5 and spindle microtubules indicated that Eg5 proteins were associated with spindle microtubules and showed a dynamic localization pattern during cell division (Fig. S1a–c).

S-Trityl-L-cysteine (STLC), a specific Eg5 inhibitor, can tightly bind to Loop 5 and inhibit the ATPase activity of Eg5 [23–25]. Dimethylenastron can selectively block the conformational change of the ADP binding pocket and significantly decrease the ADP release rate of Eg5 [26, 27]. Thus, these two specific Eg5 inhibitors are useful tools to study the functions and mechanisms of Eg5 in development. We found that the spindle assembly of HeLa cells was significantly disrupted after Eg5 inhibition by STLC and Dimethylenastron for 24 h, respectively (Fig. S1d). The percentage of mitotic cells increased to 31.36% in the 1 μM STLC group, and 59.33% in the Dimethylenastron group compared with 7.33% in the control group (Fig. S1e). Furthermore, the percentage of cells with the monopolar spindle at prometaphase and metaphase also significantly increased to 14.12% in the 1 μM STLC group and 34.30% in the 1 μM Dimethylenastron group compared with 0.00% in the control group (Fig. S1f).

We also found that Eg5 inhibition resulted in an increase in the chromosome bridge and a decrease in telophase cells (Fig. S2a, b). Eg5 inhibition led to a significant increase in the dividing cells and a decrease in the interphase cells, suggesting that Eg5 inhibition disrupts the cell cycle progression of HeLa cells (Fig. S2c–f). Taken together, these results indicate that Eg5 inhibition results in the collapse of bipolar spindle and metaphase arrest, which contribute to the increase of mitotic cells and the formation of monopolar spindles in dividing cells.

### Eg5 inhibition resulted in cell cycle arrest and the increase of polyploidy cells

To investigate the effects of Eg5 inhibition on cell cycle progression, we performed flow cytometry to study the cell cycle of HeLa cells (Fig. S3a). We found that the percentage of the G_0_–G_1_ phase cells was significantly decreased from 60.36 ± 1.24% in the control group to 32.69 ± 6.10% in the 24 h STLC group and 45.20 ± 1.49% in the 48 h STLC group (Fig. S3b). The percentage of the S phase cells was not influenced after Eg5 inhibition by 1.0 μM STLC for 24 or 48 h, respectively (Fig. S3c). Notably, the percentage of the G_2_-M phase cells was dramatically increased from 19.05 ± 0.54% in the control group to 36.29 ± 5.38% in the 24 h STLC group, and 31.89 ± 0.83% in the 48 h STLC group (Fig. S3d). We also found that the percentage of the apoptotic cells and the polyploid cells were increased after Eg5 inhibition in HeLa cells (Fig. S3e, f). These results indicate that Eg5 inhibition led to the G_2_-M phase arrest and the disruption of the cell cycle in HeLa cells. Moreover, Eg5 inhibition also causes the increase of apoptotic cells and polyploid cells in the cell cycle.

To further study the phenotype of polyploid cells in the Eg5 inhibition HeLa cells, we performed the karyotype analysis of chromosome spread. We found that STLC and Dimethylenastron-mediated Eg5 inhibition resulted in an increase in chromosome numbers, which contributes to the formation of polyploid cells (Fig. S3g). The number of chromosomes per cell increased from 60.93 ± 2.67 in the control group to 65.6 ± 3.76 in the STLC group, and 79.89 ± 4.84 in the Dimethylenastron group (Fig. S3h). In addition, the chromosomes became significantly shorter after Eg5 inhibition (Fig. S3g). We usually observed condensed and shorter chromosomes after Eg5 inhibition (Fig. S3g), which most likely originated because of the long metaphase arrest caused by the formation of monopolar spindles after Eg5 inhibition. Taken together, these results indicate that Eg5 inhibition resulted in the formation of monopolar spindle and cell cycle arrest, which finally leads to the increase of polyploid cells, the increase of apoptotic cells, and chromosome instability (Fig. S3i).

### Eg5 inhibition activated the immune responses in the mouse spleen and liver

**Fig. 1:**
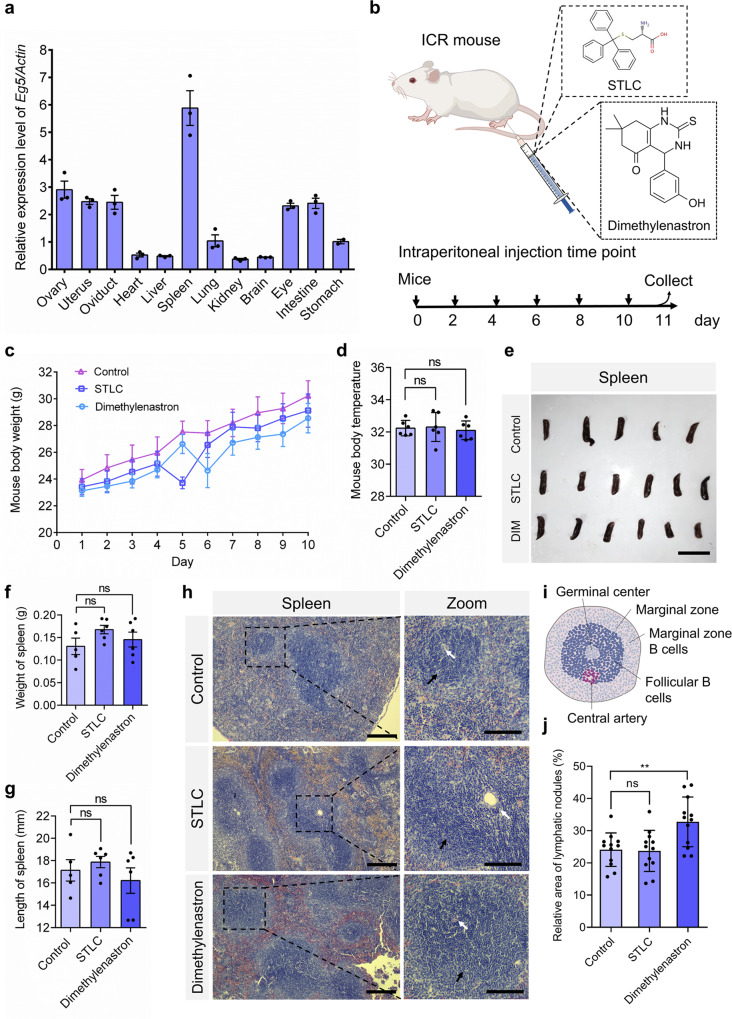
Eg5 inhibition led to the increase of lymphocytes in the spleen and the activation of immune responses in mouse models. **a** Quantitative real-time PCR analysis of relative expression levels of the *Eg5* gene in multiple organs, including the ovary, uterus, oviduct, heart, liver, spleen, lung, kidney, brain, eye, intestine, and stomach in mice. *β-Actin* served as the loading control. **b** The construction of Eg5 inhibition mouse models. The 4-week-old ICR mice were intraperitoneally injected with DMSO, STLC, and Dimethylenastron every two days for a total of five times, respectively. **c** Mouse body weight in the control, STLC, and Dimethylenastron groups were shown. The X-axis indicates the day after injection. The *Y*-axis indicated mouse body weights. **d** Mouse body temperature in the control, STLC, and Dimethylenastron groups. The *Y*-axis indicates the mouse’s body temperature (°C). **e** Representative images of mouse spleen in the control, STLC, and Dimethylenastron groups. Scale bar, 2 cm. **f** The weight of mouse spleen in the control, STLC, and Dimethylenastron groups. Control, 0.13 ± 0.02 g; STLC, 0.17 ± 0.01 g; Dimethylenastron, 0.15 ± 0.02 g. Control, *N* = 5; STLC, *N* = 6; DIM, *N* = 6. **g** The length of the spleen in the control, STLC, and Dimethylenastron groups. Control, 17.13 ± 0.96 mm; STLC, 17.87 ± 0.51 mm; Dimethylenastron, 16.21 ± 1.14 mm. Control, *N* = 5; STLC, *N* = 6; Dimethylenastron, *N* = 6. DIM indicates Dimethylenastron. **h** Representative HE staining of mouse spleen in the DMSO, STLC, and Dimethylenastron groups. The black arrow indicates the secondary lymphoid nodule, white arrow indicates the germinal center. Scale bar, 200 μm. In the zoom, scale bar, 100 μm. **i** The structure of the secondary lymphoid nodule and germinal center in mouse spleen. **j** Relative area of lymphatic nodules in the control, STLC, and Dimethylenastron groups. The lymphoid nodules with active immune responses can be divided into the dark zone and the light zone. Relative area (area of lymphatic nodule)/ (area of the spleen) in the spleen sections was measured using the Image J software. Control, 24.13 ± 1.50%, group = 12; STLC, 23.74 ± 1.84%, group = 12; Dimethylenastron, 32.76 ± 2.23%; group = 12. The group indicates the number of spleen tissues from at least 5 mice. For all graphs, mean ± SEM was shown. Student’s *t* test. ns, *p* > 0.05; **p < 0.01.

We then used quantitative real-time PCR to examine expression patterns of the *Eg5* gene in multiple tissues of mice. We found that the *Eg5* gene was highly expressed in the spleen, eye, ovary, and intestine organs (Fig. 1a). In addition, the *Eg5* gene was also widely expressed in the heart, liver, lung, kidney, brain, and stomach at a relatively low level (Fig. 1a). To further investigate the developmental roles of Eg5 in mice, we constructed the Eg5 inhibition mouse models using the specific inhibitor STLC and Dimethylenastron (Fig. 1b). The 4-week-old ICR mice were injected with STLC or Dimethylenastron by intraperitoneal injection for a total of five times every two days (Fig. 1b). We observed that the mouse body weight increased slower in the STLC and Dimethylenastron groups compared with the control group (Fig. 1c). And there were no differences in mouse body temperature among the control, STLC and Dimethylenastron groups (Fig. 1d). The mouse spleen tissues were harvested and analyzed. We found that the morphology and the weight of the spleen were not significantly influenced after Eg5 inhibition (Fig. 1e–g).

The white pulp is mainly composed of dense lymphocytes and is required for immune response. Strikingly, we found that Eg5 inhibition resulted in immune responses in the spleen of the STLC and Dimethylenastron groups (Fig. 1h, i). We observed that the periarterial lymphatic sheath of the spleen was thickened, and the lymphatic nodules in the white pulp were also increased and thickened (Fig. 1h–j). Meanwhile, the obvious dark and bright areas were increased in the lymph nodes after Eg5 inhibition (Fig. 1h). In contrast, there is no obvious immune response in the lymph nodes of the control group (Fig. 1h–j).

**Fig. 2:**
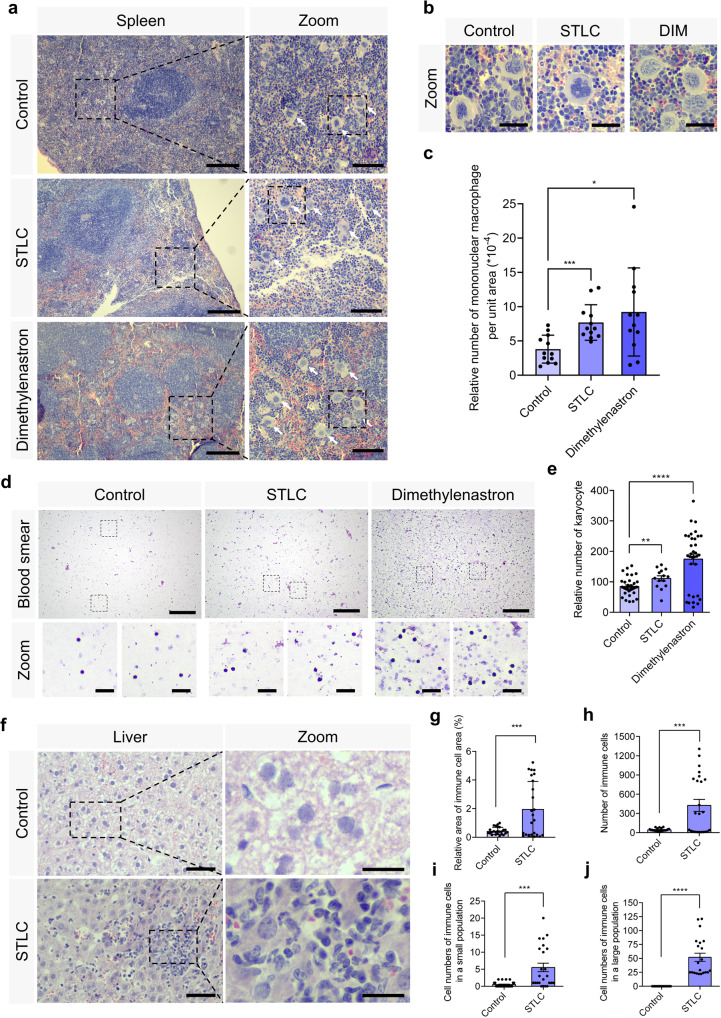
Eg5 inhibition resulted in the proliferation of monocytes in the spleen and the increase of karyocytes in the blood. **a** Representative HE staining of mouse spleen in the control, STLC, and Dimethylenastron groups. Scale bar, 200 μm. In the zoom, scale bar, 100 μm. Arrows indicate the mononuclear macrophage. **b** The enlarged images of the mononuclear macrophage were shown. Scale bar, 30 μm. **c** The relative number of mononuclear macrophages per unit area (cell number of mononuclear macrophages/ the area of spleen section) in the spleen of the control, STLC, and Dimethylenastron groups. Control, 3.80 ± 0.59; STLC, 7.70 ± 0.75. Dimethylenastron, 9.22 ± 1.86. Control, group = 12; STLC, group = 12; Dimethylenastron, group = 12. The group indicates the number of spleen tissues from at least 5 mice. **d** Representative Giemsa staining of the karyocytes in the control, STLC, and Dimethylenastron groups. Scale bar, 200 μm. In the zoom, scale bar, 30 μm. Karyocytes consist of leukocytes, lymphocytes, monocytes, plasma cells, and macrophages. The number of karyocytes in a certain area (10^6^ μm^2^) of blood smear was measured using the Image J software. **e** The relative number of karyocytes in the control, STLC, and Dimethylenastron groups. Control, 84.94 ± 4.99; STLC, 111.10 ± 8.60. Dimethylenastron, 174.60 ± 16.55. Control, group = 35; STLC, group = 14; Dimethylenastron, group = 33. **f** Representative images of mouse liver in the control, STLC, and Dimethylenastron groups. Scale bar, 50 μm. In the zoom, scale bar, 20 μm. **g** Relative area of immune cells (area of immune cells)/ (area of the liver cells). Control, 0.44 ± 0.05%; STLC, 1.98 ± 0.39%. Control, group = 25; STLC, group = 24. **h** The number of immune cells. Control, 43.68 ± 4.27; STLC, 425.60 ± 92.44. Control, group = 25; STLC, group = 24. **i** Cell numbers of immune cells in a small population. Control, 0.48 ± 0.15; STLC, 5.50 ± 1.24. Control, group = 25; STLC, group = 24. **j** Cell numbers of immune cells in a large population. Control, 0.00 ± 0.00; STLC, 51.95 ± 7.54. Control, group = 22; STLC, group = 21. The number of immune cells in a large population is defined as cell numbers of more than 20. The group indicates the number of tissues from at least 5 mice. For all graphs, mean ± SEM was shown. Student’s *t* test. *p < 0.05; **p < 0.01; ***p < 0.001; ****p < 0.0001.

The red pulp is mainly composed of splenic sinusoids and splenic cords. The blood flow in red pulp is slow, which is responsible for the interactions between the antigens and phagocytic cells. We found that there was a significant increase in the mononuclear macrophages in the STLC and Dimethylenastron groups compared with the control group (Fig. 2a–c). The number of mononuclear macrophages per unit area significantly increased after Eg5 inhibition (Fig. 2c). Furthermore, the immunofluorescence of Eg5 and α-tubulin revealed that there were monopolar spindles in the spleen cells after Eg5 inhibition (Fig. S4a, b). In addition, the TUNEL analysis indicated that the TUNEL-positive cells significantly increased after Eg5 inhibition in the spleen (Fig. S4c, d).

To further investigate the immune response after Eg5 inhibition, we examined the phenotypes of blood smears. Consistent with the results in the spleen, we observed that the number of karyocytes was dramatically increased in the STLC and Dimethylenastron groups compared with the control group (Fig. 2d, e). Taken together, the long-term Eg5 inhibition results in an increased number of monocytes, which is essential for both innate immunity and specific immunity in the body.

Furthermore, we also examined the morphology of the liver and found that there were no obvious changes in the weight, length, and width of the liver after Eg5 inhibition (Fig. S5a–d). Interestingly, we found that there was an abnormal accumulation of immune cells in the liver after Eg5 inhibition (Fig. 2f–j). The area and number of the immune cells were increased after Eg5 inhibition (Fig. 2f–h). We found that the immune cells can be characterized into two populations, including the large population and the small population. We defined the cell number of immune cells less than 20 as a small population. We observed that the populations of immune cells were significantly increased after Eg5 inhibition (Fig. 2f, i, j; Fig. S5e–g). Moreover, the number of multinucleated cells and liver cells was decreased in the STLC and Dimethylenastron groups (Fig. S5h–j). Taken together, these results indicate that Eg5 inhibition also leads to the increase of immune cells in the liver. Eg5 inhibition leads to a reduced number of liver cells, suggesting a growth inhibition in the liver after Eg5 inhibition.

In addition, we also examined the effects of Eg5 inhibition on the development of mouse kidneys (Fig. S6). We observed that the weight, length, and width of the kidneys were not influenced after Eg5 inhibition (Fig. S6a–d). We found that the relative number of glomeruli per unit area was decreased after Eg5 inhibition (Fig. S6e–h). Moreover, the number of cells in the mouse glomerulus was also decreased after Eg5 inhibition (Fig. S6g, i). These results indicate that Eg5 is also responsible for the normal development of the kidneys.

### Eg5 was essential for retinal development in both mouse and zebrafish models

**Fig. 3:**
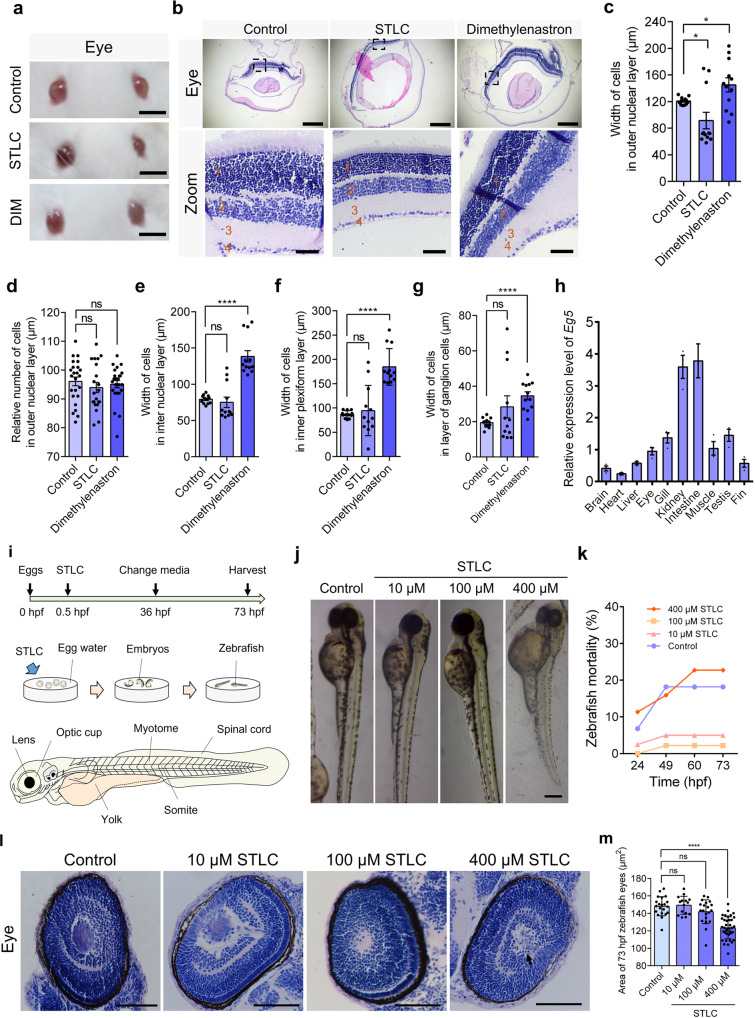
Eg5 was required for the development of the eye in mouse and zebrafish models. **a** Representative images of the mouse eyes in the control, STLC, and Dimethylenastron groups. Scale bar, 5 mm. **b** Representative HE staining of mouse eye in the control, STLC, and Dimethylenastron groups. Scale bar, 200 μm. In the zoom, scale bar, 20 μm. Symbol 1 indicates the outer nuclear layer, 2 indicates the inter nuclear layer, 3 indicates the inner plexiform layer, and 4 indicates the layer of the ganglion cells. **c** The width of cells in the outer nuclear layer. Control, 120.80 ± 1.61 μm; STLC, 91.48 ± 12.38 μm; Dimethylenastron, 145.0 ± 10.60 μm. Group = 12. **d** The number of cells in the outer nuclear layer. Control, 96.08 ± 1.51; STLC, 93.95 ± 1.76; Dimethylenastron, 95.13 ± 1.27. Group = 6. **e** The width of cells in the inter nuclear layer. Control, 79.17 ± 1.66 μm; STLC, 75.00 ± 7.11 μm; Dimethylenastron, 138.40 ± 7.80 μm. **f** The width of cells in the inner plexiform layer. Control, 86.78 ± 2.12 μm; STLC, 94.68 ± 14.91 μm; Dimethylenastron, 184.60 ± 10.89 μm. Group = 12. **g** The width of cells in the layer of the ganglion cells. Control, 19.35 ± 0.80 μm; STLC, 28.33 ± 6.23 μm; Dimethylenastron, 34.59 ± 2.26 μm. **h** Quantitative real-time PCR analysis of expression levels of the *Eg5* gene in zebrafish tissues, including the brain, heart, liver, eye, gill, kidney, muscle, testis, and fin. *β-Actin* served as the loading control. **i** The construction of the zebrafish model. STLC was added to the egg water at 0.5 hpf. The organs of zebrafish at 72 hpf were shown, including the lens, the optic cup, the myotome, the spinal cord, the somite, and the yolk. **j** Representative images of zebrafish at 73 hpf in the control and STLC groups. Scale bar, 0.2 mm. **k** The mortality of zebrafish in the control and STLC groups at 24, 49, 60 73 hpf. **l** Representative HE images of the eye in the zebrafish in the control, 10 μM STLC, and 100 μM STLC groups. Scale bar, 50 μm. **m** The area of 73 hpf zebrafish eyes (μm^2^) in the control and STLC groups. Mean ± SEM was shown. Student’s *t* test. ns, *p* > 0.05; *p < 0.05; ****p < 0.0001.

To study the developmental functions of Eg5 proteins in retinal development, we harvested the mouse eyes for further analysis. We found that Eg5 inhibition influenced retinal development in mice (Fig. 3a, b). The width of cells in the outer nuclear layer was increased after Eg5 inhibition (Fig. 3c). The relative number of cells in the outer nuclear layer was not influenced after Eg5 inhibition (Fig. 3d). Especially, the width of cells in the inter nuclear layer, the inner plexiform layer, and the layer of ganglion cells are increased in the Dimethylenastron group (Fig. 3e–g).

To further investigate the developmental roles of Eg5, we performed the genetic and evolutionary analyses of human, mouse, and zebrafish *Eg5* genes using the bioinformatics techniques (Figs. S7a–c, S8; Tables S1, S2). We found that the Eg5 proteins were highly conserved among the N-terminal motor domain through the multiple sequence alignments, suggesting a conserved role of Eg5 in the development of diverse model organisms (Fig. S8; Table S2). We then selected *Danio rerio* (zebrafish) as one of our model organisms due to its advantages in rapid embryonic development, transparent embryos, and similar genes to humans [28, 29]. We found that the *Eg5* gene is widely expressed in multiple organs in zebrafish (Fig. 3h). We treated the fertilized eggs of zebrafish AB lines with a series of concentrations of STLC at 0.5 hpf and constructed the Eg5-inhibition zebrafish models (Fig. 3i, j). According to our continuous observation and statistics, Eg5 inhibition led to slowing down cell division and the reduced number of embryonic cells in zebrafish embryos (Fig. S8a–d). We found that increasing inhibitor concentrations resulted in increased phenotypic severity (Fig. 3j, k; Fig. S9). In addition, the zebrafish mortality was not influenced in the presence of 10 μM and 100 μM STLC, but was increased in the presence of 400 μM STLC (Fig. 3k). These results suggest that Eg5 inhibition results in a slowing of embryonic cell division and a reduction in cell number in development.

We found that the retinal development was slightly disrupted in the STLC groups. Eg5 inhibition results in the disorganization of cells and the increase of apoptotic cells in the eye (Fig. 3l, m). Meanwhile, the width of cells in the visual cell layer, the bipolar cell layer, and the haversian system was not influenced in the STLC groups compared with the control (Fig. S9g–i). Especially, the area of zebrafish eyes was smaller in the 400 μM STLC groups compared with the control group (Fig. 3m), suggesting a growth inhibition in the retinal cells after Eg5 inhibition.

### Eg5 inhibition influenced the somite formation, the head development, and the maintenance of kidneys during organogenesis

**Fig. 4:**
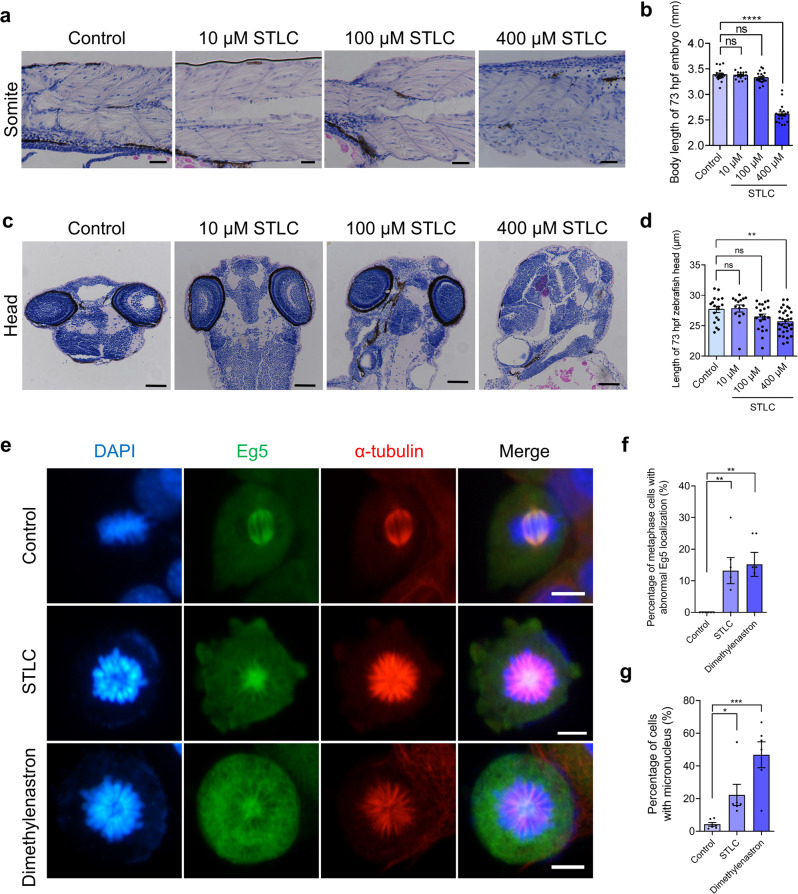
Eg5 is essential for cell division of somite cells and fibroblasts, and the development of head and somite. **a** Representative HE images of the zebrafish somites at 73 hpf in the control, 10 μM STLC, and 100 μM STLC groups. Scale bar, 50 μm. **b** The body length of 73 hpf zebrafish embryos in the control, 10 μM STLC, and 100 μM STLC groups. **c** Representative HE images of the zebrafish head at 73 hpf in the control, 10 μM STLC, and 100 μM STLC groups. Scale bar, 100 μm. **d** The length of 73 hpf zebrafish heads in the control, 10 μM STLC, 100 μM STLC, and 400 μM STLC groups. **e** Representative immunofluorescence images of Eg5 and α-tubulin. DAPI, blue; Eg5, green; α-tubulin, red. NIH/3T3 cells were incubated with STLC and Dimethylenastron for 48 h. Scale bar, 10 μm. **f** The percentage of metaphase cells with abnormal Eg5 localization in the Control, STLC, and Dimethylenastron groups. Control, 0.00 ± 0.00%; 1 μM STLC, 13.20 ± 4.13%; 1 μM Dimethylenastron, 15.18 ± 3.79%. Groups = 6, *N* = 306, 56, 40. **g** The percentage of cells with micronucleus in the Control, STLC, and Dimethylenastron groups. Control, 4.27 ± 1.16%; 1 μM STLC, 22.24 ± 6.50%; 1 μM Dimethylenastron, 46.76 ± 7.91%. Groups = 6, *N* = 306, 67, 63. For all graphs, mean ± SEM was shown. Student’s *t* test. ns, *p* > 0.05; *p < 0.05; **p < 0.01; ***p < 0.001, ****p < 0.0001.

Strikingly, we found that the somite formation of zebrafish was significantly influenced after Eg5 inhibition (Fig. 3j; Fig. S9a–e). HE staining results indicated that the proliferation of somite cells was significantly suppressed after Eg5 inhibition (Fig. S9e, f). We also found that the cell organization was significantly disrupted after Eg5 inhibition in the zebrafish somites at 73 hpf (Fig. 4a). Meanwhile, the body length of zebrafish was also decreased after Eg5 inhibition (Fig. 4b).

Moreover, we found that Eg5 inhibition resulted in the malformation of the head and the disorganization of craniofacial cells (Fig. 4c, d), which were similar to the human microcephaly phenotype in the previous studies [21, 30, 31]. Notably, there was an increased number of apoptotic cells in the head of the STLC groups. And the length of the zebrafish head was decreased after Eg5 inhibition (Fig. 4d).

**Fig. 5:**
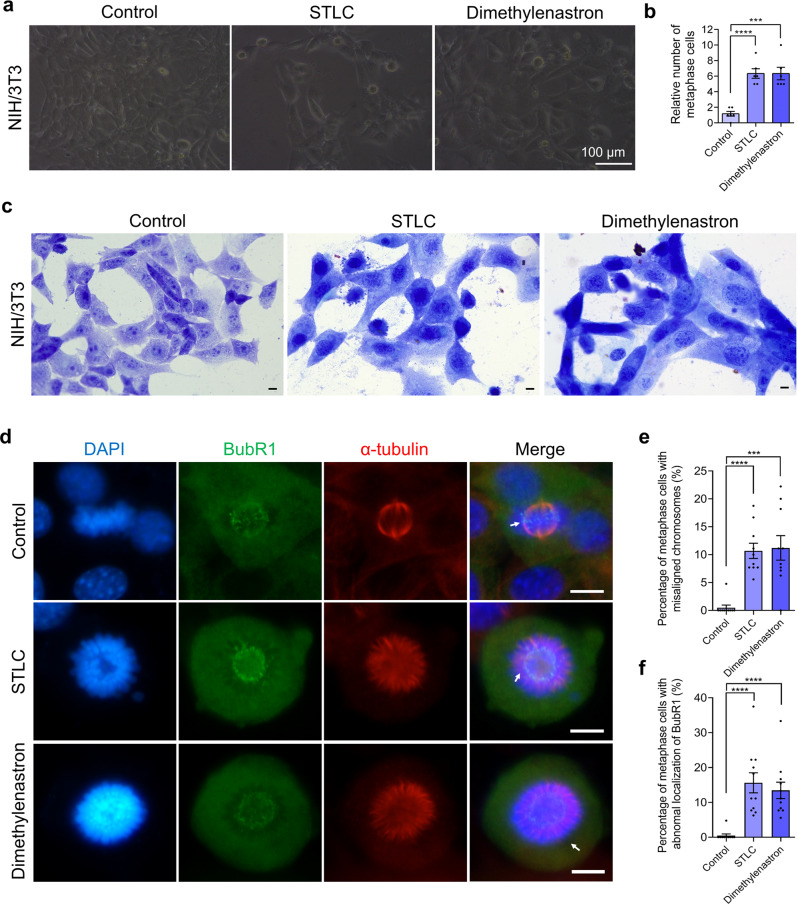
Eg5 inhibition resulted in abnormal localization of BubR1 and the formation of the monopolar spindle. **a** Representative image of NIH/3T3 cells in the Control, 1 μM STLC, and 1 μM Dimethylenastron groups. Scale bar, 100 μm. NIH/3T3 cells were incubated with STLC and Dimethylenastron for 48 h. **b** The relative number of metaphase cells in the control, STLC, and Dimethylenastron. Control, 1.67 ± 0.31; 1 μM STLC, 6.33 ± 0.61; 1 μM Dimethylenastron, 6.33 ± 0.80. Groups = 6. **c** The Giemsa staining of NIH/3T3 cells after being treated with STLC and Dimethylenastron for 48 h. Scale bar, 10 μm. **d** Immunofluorescence images of BubR1 and α-tubulin in NIH/3T3 cells during metaphase. DAPI, blue; BubR1, green; α-tubulin, red. Scale bar, 10 μm. **e** The percentage of metaphase cells with misaligned chromosomes in the control, 1 μM STLC, and 1 μM Dimethylenastron groups. Control, 0.48 ± 0.48%; STLC, 10.68 ± 1.36%; Dimethylenastron, 11.20 ± 2.20%. Groups = 10. *N* = 352, 112, 131. **f** The percentage of metaphase cells abnormal localization of BubR1 in the control, STLC, and Dimethylenastron. Control, 0.48 ± 0.48%, Groups = 10, *N* = 352; STLC, 15.62 ± 2.87%, Groups = 11, *N* = 120; Dimethylenastron, 13.44 ± 2.35%, Groups = 11, *N* = 143. For all graphs, mean ± SEM was shown. Student’s *t* test. ***p < 0.001, ****p < 0.0001.

To further analyze molecular mechanisms of Eg5 in the proliferation of somite cells, we utilized the mouse embryo fibroblast NIH/3T3 cell line as one of our model cells. We found that the localization of Eg5 proteins enriched in spindle microtubules of NIH/3T3 changed significantly in the metaphase cells after Eg5 inhibition by STLC and Dimethylenastron for 48 h (Fig. 4e–f). Eg5 inhibition resulted in metaphase-arrested, chromosome misalignment, and micronucleus. The percentage of cells with micronucleus increased to 22.24% in the 1 μM STLC group, and 46.76% in the Dimethylenastron group compared with 4.27% in the control group (Fig. 4g). In the mouse embryo fibroblast NIH/3T3 cells, Eg5 inhibition resulted in an increased number of metaphase cells (Fig. 5a–c). And the Giemsa staining results indicated that apoptotic cells were induced by STLC and Dimethylenastron treatment (Fig. 5c). In addition, we examined the localization of BubR1 protein, a key checkpoint kinase in the spindle assembly checkpoint, and found that Eg5 inhibition significantly affected the localization of BubR1 proteins, which were translocated to misaligned kinetochores (Fig. 5d–f). The percentage of metaphase cells with abnormal localization of BubR1 proteins increased to 15.62% in the 1 μM STLC group, and 13.44% in the Dimethylenastron group compared with 0.48% in the control group (Fig. 5f). Taken together, these results indicate that Eg5 is required for the regulation of spindle assembly, chromosome alignment, and spindle assembly checkpoint. The changes in Eg5 and BubR1 are secondary effects of the changes to the spindle and chromosomes after Eg5 inhibition. Eg5 inhibition results in the suppression of cell growth and the activation of the spindle assembly checkpoint, which is responsible for growth retardation and multi-organ diseases in development.

## Discussion

Kinesin-5 motors crosslink microtubules, generate outward pushing force on centrosomes, and regulate centrosome separation [5, 10]. Eg5 heterodimers mediate the formation of bipolar spindle and the alignment of chromosomes [32, 33]. Eg5 ablation results in mitotic spindle defects, the activation of spindle assembly checkpoint, and cell apoptosis [16]. In this study, we have shown that Eg5 inhibition results in the collapse of the bipolar spindle, chromosome misalignment, and the activation of the spindle assembly checkpoint during cell division. Furthermore, long-term inhibition of Eg5 significantly increases the formation of polyploid and apoptotic cells in multiple organs.

The *Eg5* gene is widely expressed in multiple organs during murine development and is highly expressed in proliferating tissues [34, 35]. Our data also suggest that the *Eg5* gene is highly expressed in the spleen and widely expressed in multiple organs in mice. In mice, homozygous deletion of Eg5 results in embryonic lethality and defects in the proliferation at the early stage of embryogenesis [34, 35]. In zebrafish embryos, the whole-mount in situ hybridization assays have shown that the *Eg5* gene is strongly expressed in the proliferating tissues of the embryos, including various parts of the brain, the spinal cord, and the retinal cells [19]. In this study, we have also found that the *Eg5* gene is widely expressed in multiple tissues in zebrafish, suggesting a potential common role in multiple organs.

The spleen is the second-largest lymphoid organ in the body. Dendritic cells, macrophages, NK cells, and various monocytes in the spleen first initiate innate immune responses to apoptotic cells [36]. There are also many kinds of white blood cells in the spleen, including T cells, B cells, dendritic cells, and different subpopulations of macrophages [37]. When apoptotic cells appear in the body, dendritic cells are responsible for collecting presentation antigens in peripheral tissues and migrating to the spleen lymph nodes under the action of chemokines to activate the native T cells [36, 38]. Activated T cells proliferate and differentiate into CD4^+^ T cells and CD8^+^ T cells to participate in further cellular immunity [39]. Strikingly, we found that Eg5 inhibition results in the accumulation of polyploid and apoptotic cells, which can stimulate the innate and adaptive immune response.

**Fig. 6:**
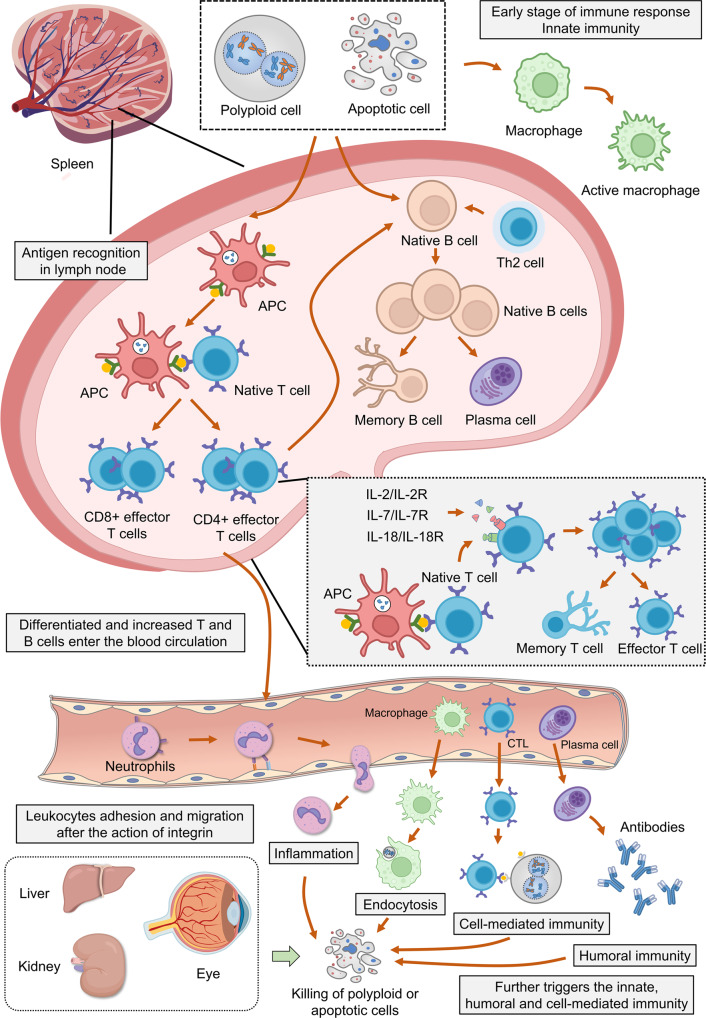
Schematic diagram of the immune response and developmental processes after Eg5 inhibition. Eg5 inhibition results in the collapse of the bipolar spindle, metaphase arrest, and the activation of the spindle assembly checkpoint, which further leads to the increase of polyploid and apoptotic cells. First, the polyploid and apoptotic cells are recognized and phagocytosed by the macrophages during the innate immunity. Secondly, the polyploid and apoptotic cells stimulate the proliferation and differentiation of lymphoid T and B cells during adaptive immunity. This process is mediated by antigen-presenting cells (APCs) followed by the activation of T cells. In addition, IL-2, IL-7, and IL-18 also stimulate T cells to proliferate and differentiate into memory T cells and effector T cells. Moreover, B cells are also activated by Th2 cells and CD4^+^ cells to proliferate and differentiate into memory B cells and plasma cells. During blood circulation, the differentiated T and B cells, neutrophils, and cytotoxic T lymphocytes (CTLs) adhere, migrate and enter other organs to recognize and kill polyploid and apoptotic cells throughout the body. After Eg5 inhibition, the immune responses, including inflammation, endocytosis, cell-mediated immunity, and humoral immunity, occur in the organs with vigorous metabolism and cell division, such as the liver, kidney, and eyes.

B cells in the spleen are mainly divided into two subpopulations: marginal zone B cells and follicular B cells. When exposed to antigens, marginal band B cells can capture antigens through complement receptors, further promoting cellular immunity [40]. Follicular B cells rapidly circulate between the bright and dark areas of the germinal center. During the humoral immune response, follicular B cells undergo rapid proliferation, class switching, and somatic hypermutation during this process [41, 42]. In this study, we have revealed that Eg5 inhibition results in cell cycle arrest and the formation of apoptosis cells, which further triggers immune responses in the body, including the innate and adaptive immune responses (Fig. 6). Polyploidy could in part directly trigger immune response through genomic stress and eventually lead to cell death and the release of cell content, which could also indirectly trigger an immune response. However, direct evidence for these hypotheses is still missing and we also cannot exclude the possibility that the drugs could have triggered an immune response independently of its effect on Eg5.

In human patients, the individuals with mutations of *Eg5* show severe microcephaly, bilateral chorioretinopathy, and developmental delay, including a thin body [21] and growth retardation [22]. Lymphedema in patients is present at birth and is usually restricted to both lower limbs [19, 20]. Mutagenic screens of zebrafish have revealed that the *Eg5* gene is critical for the normal development of secondary motor neurons and oligodendrocytes. Glial cells were arrested in metaphase followed by apoptotic cell death [43]. In both *Drosophila* and humans, excess Tau proteins influence kinesin-5 Klp61F to mediate aneuploidy and cell death during the neurodegenerative, suggesting an evolutionarily conserved role of kinesin-5 motors in development [44].

Microcephaly with or without chorioretinopathy, lymphedema, or mental retardation (MCLMR) syndrome is a rare autosomal dominant genetic disease (MIM No.152950) featured by different degrees of microcephaly, eye defects including chorioretinopathy, congenital lymphedema, and intellectual disability [20, 45]. Previous studies have identified 27 carriers of 14 mutant *Eg5* alleles in 15 independent families and found that *Eg5* mutation is a significant proportion of cases of MLCRD (microcephaly, primary lymphedema, and chorioretinal dysplasia) syndrome and CDMMR (chorioretinal dysplasia, microcephaly, and mental retardation) syndrome [19]. The reports of clinical features of 37 individuals from 22 families with *Eg5* mutations have shown that 22 probands had microcephaly, followed by slow head growth and worsening head circumference after birth [20]. In addition, the substantial degrees of phenotype variability might be related to multiple functions of Eg5 in mitosis and in vesicle transport and cytoplasmic roles in non-dividing cells, which remains to be clarified in the future.

Chorioretinopathy is a highly specific feature seen in 59% of patients with Eg5 mutations [19]. Other ophthalmological findings, including hypermetropia and hypermetropic astigmatism, myopia and myopic astigmatism, bilateral retinal folds, microphthalmia, and other ocular features, were found in 78% of Eg5 mutants [20]. Previous studies have also identified widespread retinal pigment epithelial degeneration, retinal atrophy, and pigment clumping in patients with Eg5 mutations [21]. Further imaging and electroretinography results have shown a borderline low retinal thickness and retina-wide photoreceptor dysfunction in the patients [46].

Five major genes, including *NDP* (OMIM No.300658), *FZD4* (OMIM No.604579), *LRP5* (OMIM No.603506), *TSPAN12* (OMIM No.613138) and ZNF408 (NCBI No.79797) are essential for half of the cases of autosomal dominant familial exudative vitreoretinopathy (FEVR) [30, 47–51]. Furthermore, Eg5 mutations were gradually discovered as a common cause of FEVR from the avascular zone in the peripheral retina to bilateral complete retinal detachment [31, 52, 53]. However, the cellular and molecular mechanism underlying how kinesin-5 Eg5 regulates retinal vascular development remains largely unknown.

In humans, Eg5 proteins are located at the inner segments and ciliary compartments of photoreceptors and in the retinal pigment epithelium [46]. The Eg5-associated disease represents a potential system ciliopathy with defects in photoreceptor development and retinal angiogenesis [22, 46]. The *Eg5* vascular endothelial cell-specific knockout mouse models suggest that the *Eg5*-deficient retinal vascular phenotype is independent of the established Norrin Wnt β-catenin signaling pathway [54, 55]. In this study, we have demonstrated that Eg5 inhibition results in cell apoptosis in the eye, which further contributes to the disorganization of retinal cells and cell layers. We conclude that the roles of Eg5 in bipolar spindle assembly and chromosome alignment are also critical for the cell division of retinal cells and retinal development. The two inhibitors STLC and Dimethylenastron both target kinesin-5 Eg5. But there are several different effects, which may be due to the different drug binding affinity and interactional efficiency. Defects in cell division caused by Eg5 inhibition, especially the monopolar spindle and cell cycle arrest, can lead to chorioretinopathy in *Eg5* mutants in humans, mice, and zebrafish. In the future, the long-term follow-up data and further in vitro studies on the cells derived from specific tissues in the patients with *Eg5* mutations would gain insights into detailed mechanisms of Eg5 in organogenesis during development.

In this study, we found glomerular loss and a reduced number of cells in the mouse glomerulus in mouse kidneys after Eg5 inhibition. Previous studies have shown that decreased glomerular number leads to decreased glomerular filtration rate [56], followed by increased reabsorption of sodium and water, leading to the retention of sodium and water and further tissue edema [57]. These results may explain the phenotypes of lymphedema and congenital bilateral lower limb edema observed in *Eg5* mutant patients [19, 21, 30]. We consider that in patients with renal disease, the level of plasma albumin decreases due to proteinuria, and then the colloid osmotic pressure in the blood vessels decreases, and the interstitial fluid increases, resulting in edema. In the future, the specific mechanisms of lower limb edema in patients with *Eg5* deficiency can be explored through the examination of urea and creatinine in blood, and protein contents in the urine [58].

In conclusion, we have revealed that the *Eg5* gene is widely expressed in a variety of organs in both mice and zebrafish. We demonstrated that Eg5 inhibition significantly disrupts the organization of bipolar spindle and chromosome segregation, which contribute to cell cycle arrest at metaphase, the formation of multinuclei in mitosis, and subsequent apoptotic cell death. Strikingly, we found that Eg5 inhibition stimulates the immune response and the increase of lymphocytes in the spleen. The immune response may be activated by polyploid and apoptotic cells after Eg5 inhibition, which can explain the cellular basis for microcephaly, primary lymphedema, and chorioretinal dysplasia syndrome in patients with heterozygous *Eg5* mutations. We also found that Eg5 inhibition significantly influences somite formation and retinal development. Our data have provided evidence that Eg5 depletion results in defects in spindle assembly and chromosome segregation, which finally lead to multi-organ syndromes. The conserved roles of Eg5 in centrosome separation and bipolar spindle formation are required for cell division of proliferating cells and the development of multiple organs.

## Materials and methods

### Animals and animal ethics

All animal protocols were reviewed and approved by the Institutional Animal Care and Use Committee at Fujian Medical University, China (protocol number SYXK2016-0007). Animal experiments, care, and use were conducted following the guidelines of the Care and Use of Laboratory Animals of the National Institutes of Health (NIH publications No. 8023, revised 1978).

### Animal experiments, maintenance, and model construction

*Danio rerio* AB line zebrafish (Chinese Zebrafish Resource Center, Cat. CZ1) were raised and maintained in 14 h light/10 h dark conditions at 28.5 °C. Fish lines were maintained by the standard guidelines [58]. Zebrafish embryos were obtained from the natural spawning of wild-type fish and collected at different stages according to the standard guidelines [28, 29]. Pharmacological inhibition of Eg5 in zebrafish embryos was performed by adding 10, 100, or 400 μM STLC (Santa Cruz Biotechnology, Cat. sc-202799) to egg water at the one-cell stage (0.5 hpf), respectively. Zebrafish embryos were maintained in a 6-well plate (Corning) and were imaged using a light microscope (Jiangnan) and recorded with the ToupView software (Touptek Photonics).

Male and female, wild-type 4-week-old ICR mice were obtained from Shanghai SLAC Laboratory Animal co. LTD (Shanghai, China). Mice were maintained in a specific pathogen-free animal facility at Fujian Medical University accredited by the Association for Assessment and Accreditation of Laboratory Animal Care International and exposed to a 14 h light/10 h dark condition with free access to water and food.

### Cell culture, transfection, and treatment

HeLa cells (ATCC No. CCL-2) and NIH/3T3 (ATCC No. CRL-1658) were obtained from American Type Culture Collection. Cells were cultured in DMEM/high glucose (Gibco, Cat. C11995500BT) with 10% heat-inactivated fetal bovine serum (Every green, Cat. 1101–8611) and 1% penicillin/streptomycin solution (Hyclone, Cat. SV30010).

For Eg5 inhibition in cultured cells, cells were incubated with a series of concentrations of STLC (Santa Cruz Biotechnology, Cat. sc-202799) as indicated in each figure legend. For Eg5 inhibition in mice, specific inhibitors were intraperitoneally injected into the abdominal cavity of mice every two days five times. STLC (S-Trityl-L-cysteine, Santa Cruz Biotechnology, Cat. sc-202799) was injected at a final concentration of 10 μM (at the 3.6 mg/kg dose). Dimethylenastron (MedChemExpress, Cat. HY-19944) was injected at a final concentration of 5 μM (at the 3 mg/kg dose).

### Histological analysis

Animals were euthanized and harvested for histological analyses. Tissues were fixed in 10% formaldehyde at room temperature for 12 h and dehydrated in gradient ethanol, including 70% ethanol for 1 h, 85% ethanol for 1 h, 95% ethanol for 1 h, and anhydrous ethanol for 1 h. After incubating with xylene for 1 h, samples were fixed in paraffin for 1 h at 60 °C. The 5-μm-thick slides were immersed in xylene for 40 min and then incubated in 100% ethanol for 6 min, in 95% ethanol, in 90% ethanol, in 80% ethanol, and in 70% ethanol for 2 min, respectively. Samples were stained with the Mayer’s hematoxylin solution for 6 min and then incubated with 1% ethanol hydrochloride for 3 s. Slides were stained with 1% eosin for 15 s and then incubated with 95% ethanol for 5 s, with anhydrous ethanol for 2 min. After incubating with xylene for 40 min, slides were sealed with neutral gum.

### Chromosome preparation and karyotyping

Cells were cultured for 24 h and then treated with 0.1 μg/ml colchicine (Sinopharm chemical reagent, Cat. 61001563) at 37 °C for 4 h. Cells were harvested and centrifuged at 1000 × *g* for 5 min. Cells were treated with 0.075 mol/L KCl solution (pH7.0) at 37 °C for 20 min. Cells were incubated with the methanol-glacial acetic acid solution (v:v = 3: 1) for 10 min, and then dropped onto ice-cold slides from 20 to 30 cm height. The slides were baked and then stained with the Giemsa staining solution (pH6.8) for 7 min. Images were captured using a light microscope (Nikon E200) equipped with a Plan Fluor 40×/NA 0.75 objective.

### Immunohistochemistry and confocal microscopy

For immunofluorescence, cells were fixed in 4% paraformaldehyde in PBS for 10 min, washed three times with PBS, and then permeabilized with 0.25% Triton X-100/PBS for 10 min at room temperature. For antigen blocking, cells were blocked with the blocking buffer (1% BSA in PBS containing 0.05% Tween-20) for 60 min at 37 °C. Cells were incubated with primary antibodies for 12 h at 4 °C and then washed three times with PBS at room temperature. Cells were incubated with Alexa Fluor-488 or −555-conjugated secondary antibodies for 2 h at 37 °C. After washing five times with PBS, the nuclei were stained with DAPI (Beyotime, Cat. C1006). Coverslips were mounted with the mounting reagent (Beyotime, Cat. P0126).

For cell apoptosis analysis, TdT-mediated dUTP Nick-End Labeling (TUNEL) assays were performed using one-step TUNEL apoptosis assay kit (Beyotime Cat. C1086) according to the manufacturer’s protocols. The TUNEL-positive cells were imaged under fluorescent microscopy (Nikon Ti-S2) at the 488 nm excitation.

The antibodies used in this study are listed as follows: rabbit anti-TUBA4A polyclonal antibody (Sangon Biotech, Cat. D110022), mouse anti-tubulin monoclonal antibody (Beyotime, Cat. AT819), rabbit anti-Eg5 monoclonal antibody (Abcam, Cat. ab254298), mouse anti-α-tubulin monoclonal antibody (Abcam, Cat. ab7291), rabbit anti-BubR1 monoclonal antibody (Abcam, Cat. ab251326), Alexa Fluor 488-conjugated goat anti-rabbit secondary antibody (Beyotime, Cat. A0423), and Alexa Fluor 555-conjugated donkey anti-mouse secondary antibody (Beyotime, Cat. A0460).

Images were captured using a fluorescent microscope (Nikon Ti-S2) equipped with a Plan Fluor 10×/NA 0.25 objective, a Plan Fluor 20×/NA 0.40 objective, and a Plan Fluor 40×/NA 0.75 objective. Images were collected using a DS-Ri2 camera (Nikon) and NIS-Elements imaging software (Nikon). High-resolution confocal images were recorded using a laser scanning confocal microscope (Leica No. TCS SP8) equipped with a 63×/NA 1.40 water-immersion objective (Leica HC PL APO CS2). For the analyses of fluorescence intensities, the red/green/blue fluorescence intensities were quantified and analyzed by the line scan and plot analysis tool using the ImageJ software (National Institutes of Health).

### RNA isolation, cDNA synthesis, and quantitative real-time PCR

Samples were homogenized in the Trizol reagents. RNA isolation was performed via column methods according to the manufacturer’s protocols (Sangon Biotech, Cat. B511321). cDNA was synthesized using PrimeScript RT Master Mix (TaKaRa, Cat. RR036A) according to the manufacturer’s protocols. Up to 500 ng of RNA was reverse-transcribed per sample per reaction and the final volume of cDNA generated was 10 μl. The following PCR program for cDNA synthesis was used: 37 °C, 30 min; 85 °C, 5 s. For RT-qPCR, the mixtures of template DNA, specific primers, and BeyoFast SYBR Green qPCR Mix (Beyotime, Cat. D7265) were used in each reaction according to the manufacturer’s guidelines. The following conditions were used: 95 °C heat for 2 min; 95 °C, 15 s, 60 °C, 30 s for 40 cycles; 95 °C, 15 s, 60 °C, 15 s and 95 °C, 15 s. PCR programs were carried out using an AriaMx real-time PCR cycler (Agilent Technologies, No. AriaMx G8830-64001). *Ct* values from at least three duplicates were analyzed and the 2 *Ct* (target)/2 *Ct* (reference) method was applied in the calculation of the relative expression level of target genes. *β-Actin* gene was severed as a reference gene for mouse and zebrafish larvae, respectively.

The following primers were used: for gene expression analysis in mice, *Eg5* F1, 5′-TGGTGGTGAGATGCAGACCAT-3′; *Eg5* R1, 5′-CGTCAACCCTGCAGTCCGTA-3′ (*M. musculus* Eg5, GenBank accession number NM_010615.2). *β-Actin* F1, 5′-ACGATATCGCTGCGCTGGTC-3′; *β-Actin* R1, 5′-ATTCCCACCATCACACCCTGG-3′ (*M. musculus β-Actin*, GenBank accession number NM_007393.5). For gene expression analyses in zebrafish, *Eg5* F2, 5′-AGCAAGATCGGCTTAACGGT-3′; *Eg5* R2, 5′-CTGTGTTAAAGGGTCTGCATCG-3′ (zebrafish *Eg5*, GenBank accession number NM_173261.2). *β-Actin* F2, 5′-GAGCTATGAGCTGCCTGACG-3′; *β-Actin* R2, 5′-ACCGCAAGATTCCATACCCAG-3′ (*D. rerio β-Actin*, GenBank accession number NM_131031.2).

### Flow cytometry

For cell cycle analysis, cells were digested using 0.25% Trypsin-EDTA for 2 min and then centrifuged at 1000 × *g* for 5 min. Cells were fixed using 70% ethanol at 4 °C for 12 h and then centrifuged at 1000 × *g* for 5 min. Cells were incubated with propidium iodide solution (0.1% Triton X-100/PBS, 50 μg/ml PI and 20 μg/ml RNase A) at 37 °C for 1 h. The fluorescence was detected at the excitation wavelength of 488 nm using a BD FACSCantoTMII flow cytometer according to the manufacturer’s protocols. Data analysis of DNA content and light scattering were performed using the Modfit LT32 software (Verity Software House) and the FlowJo software. The X-axis PI-A and Y-axis PI-W were selected and the linearity was set at 1.85–2.00 during the setup of gate and cell cycle analysis according to the ModFit guideline (https://www.vsh.com/Documentation/ModfitLT). The shaded areas for G_0_–G_1_, G_2_–M, and S phases were determined according to the standard cell cycle analysis [59] and the ModFit LT help manual. The differences between the diploid G_2_–M cells and tetraploid G_1_ cells were discriminated according to cell size and DNA content. The apoptotic cells were scored in the region (X-axis PI-A: 0–6; *Y*-axis PI-W: 30–90) according to the manual protocols.

### Statistical analysis

All data were obtained from at least three independent experiments and were shown as mean ± SEM. When n > 4 in at least one group, center values were plotted as mean ± SEM (error bars) with individual data points superimposed. No statistical methods were used to predetermine the sample size. The experiments were not randomized and investigators were not blinded to allocation during experiments and outcome assessment. Data were analyzed by unpaired student’s *t* test using Microsoft Excel and GraphPad Prism version 6 (GraphPad Software). *P* values < 0.05 were considered statistically significant. ns, *P* > 0.05; *P < 0.05; **P < 0.01; ***P < 0.001; ****P < 0.0001. For the phylogenetic tree analysis, amino acid sequences of model organisms were obtained from the NCBI database, aligned by the ClustalW algorithm, and analyzed by the neighbor-joining method using the Mega X software.

## Supplementary information

Supplemental Material

Supplemental Table S1-S2

## Data Availability

The data that support the findings of this study are available from the corresponding author upon reasonable request.
