# Supplementary material for: Kinesin-5 Eg5 is essential for spindle assembly, chromosome stability and organogenesis in development
Source: Cell Death Discov. 2022 Dec 13;8:490. doi: 10.1038/s41420-022-01281-1 (PMC9747790; doi:10.1038/s41420-022-01281-1)
Supplement: Supplementary file 1 — Supplemental Material [file 41420_2022_1281_MOESM1_ESM.docx]

**Supplemental information**

**Kinesin-5 Eg5 is essential for spindle assembly, chromosome stability and organogenesis in development**

Wen-Xin Yu^1,2,#^, Yu-Kun Li^1,2,#^, Meng-Fei Xu^1,2,#^, Chen-Jie Xu^1,2^, Jie Chen^1,2^, Ya-Lan Wei^3,4^, Zhen-Yu She^1,2,*^

**Supplemental figures S1-9, Table S1-S2, and figure legends**

**Figure S1**

**
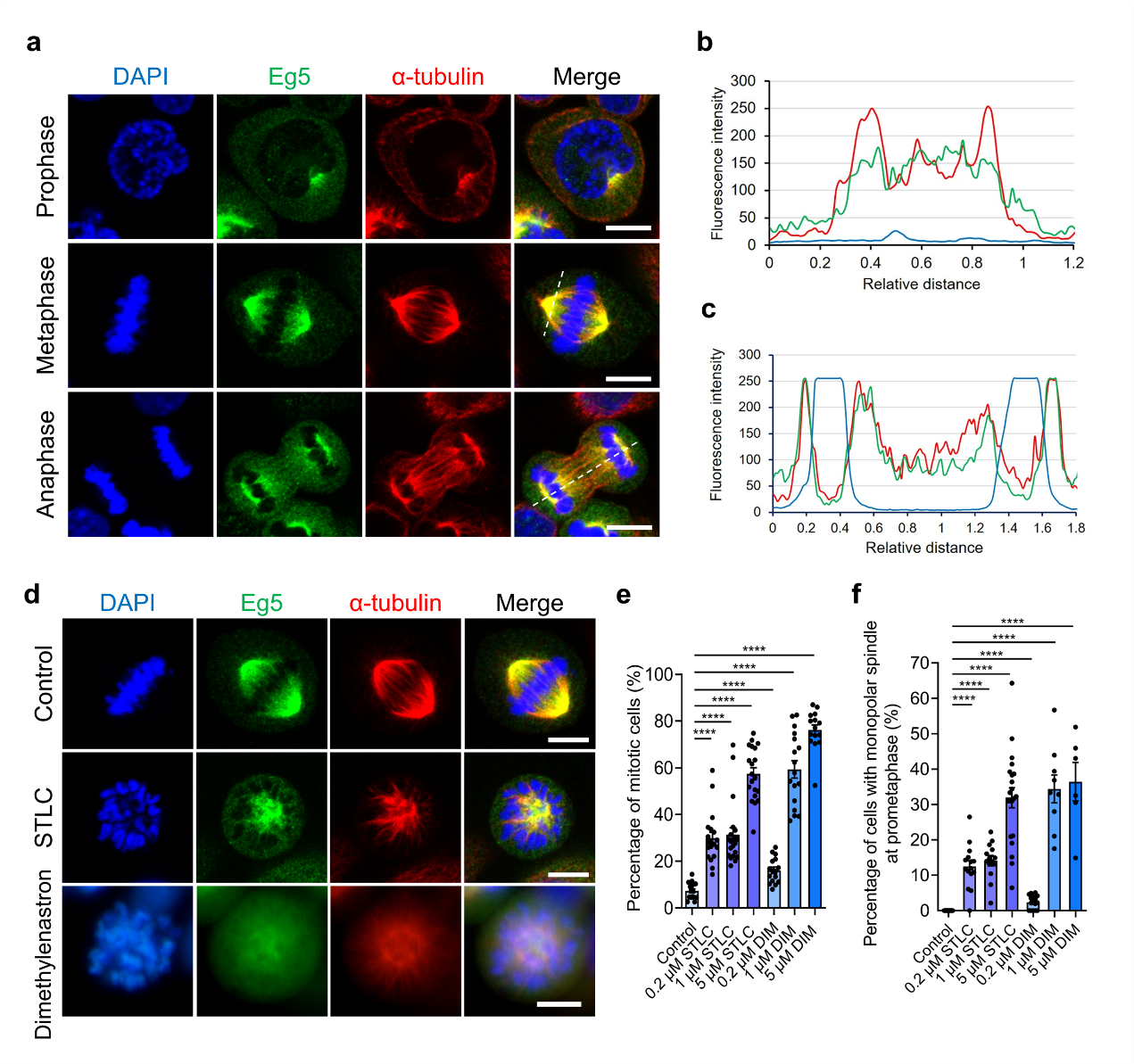
**

**Fig. S1** **Eg5 proteins located at the spindle microtubule and were required for spindle assembly in HeLa cells. a** Representative immunofluorescence images of Eg5 and α-tubulin in HeLa cells during prophase, metaphase and anaphase. DAPI, blue; Eg5, green; α-tubulin, red. Scale bar, 10 μm. **b, c** Line-scan analysis of Eg5 and α-tubulin at metaphase (b) and anaphase (c) in HeLa cells. The Y-axis indicates the fluorescence intensity. The X-axis indicates the relative distance. **d** Representative images of Eg5 and α-tubulin in the control, STLC and Dimethylenastron groups. Eg5 inhibition by 1 μM STLC or 1 μM Dimethylenastron resulted in the formation of monopolar spindle. DAPI, blue; Eg5, green; α-tubulin, red. Scale bar, 10 μm. **e** The percentage of mitotic cells in the control, STLC, and Dimethylenastron groups. HeLa cells were incubated with 0.2, 1, 5 μM STLC, or 0.2, 1, 5 μM Dimethylenastron, respectively. Control, 7.33 ± 0.84%, group = 17; 0.2 μM STLC, 29.90 ± 2.50%, group = 19; 1 μM STLC, 31.36 ± 2.95%, group = 21; 5 μM STLC, 57.46 ± 2.54%, group = 20; 0.2 μM Dimethylenastron, 16.12 ± 1.36%, group = 16; 1 μM Dimethylenastron, 59.33 ± 3.76%, group = 17; 5 μM Dimethylenastron, 76.17 ± 2.19%, group = 15. In the X-axis, DIM indicates Dimethylenastron. **f** The percentage of cells with monopolar spindle at prometaphase. Control, 0.00 ± 0.00%, group = 27; 0.2 μM STLC, 12.51 ± 1.59%, group = 15; 1 μM STLC, 14.12 ± 1.36%, group = 14; 5 μM STLC, 31.93 ± 2.84%, group = 21; 0.2 μM Dimethylenastron, 2.38 ± 0.48%, group = 16; 1 μM Dimethylenastron, 34.40 ± 3.95%, group = 9; 5 μM Dimethylenastron, 36.45 ± 5.43%, group = 6. In the X-axis, DIM indicates Dimethylenastron. In the control, STLC, Dimethylenastron group, 1000 cells were analyzed. In figure 1, the group indicates the number of cell populations (N = 60-100). For all graphs, mean ± SEM was shown. Student’s *t*-test. ****, *p* < 0.0001.

**Figure S2**

**
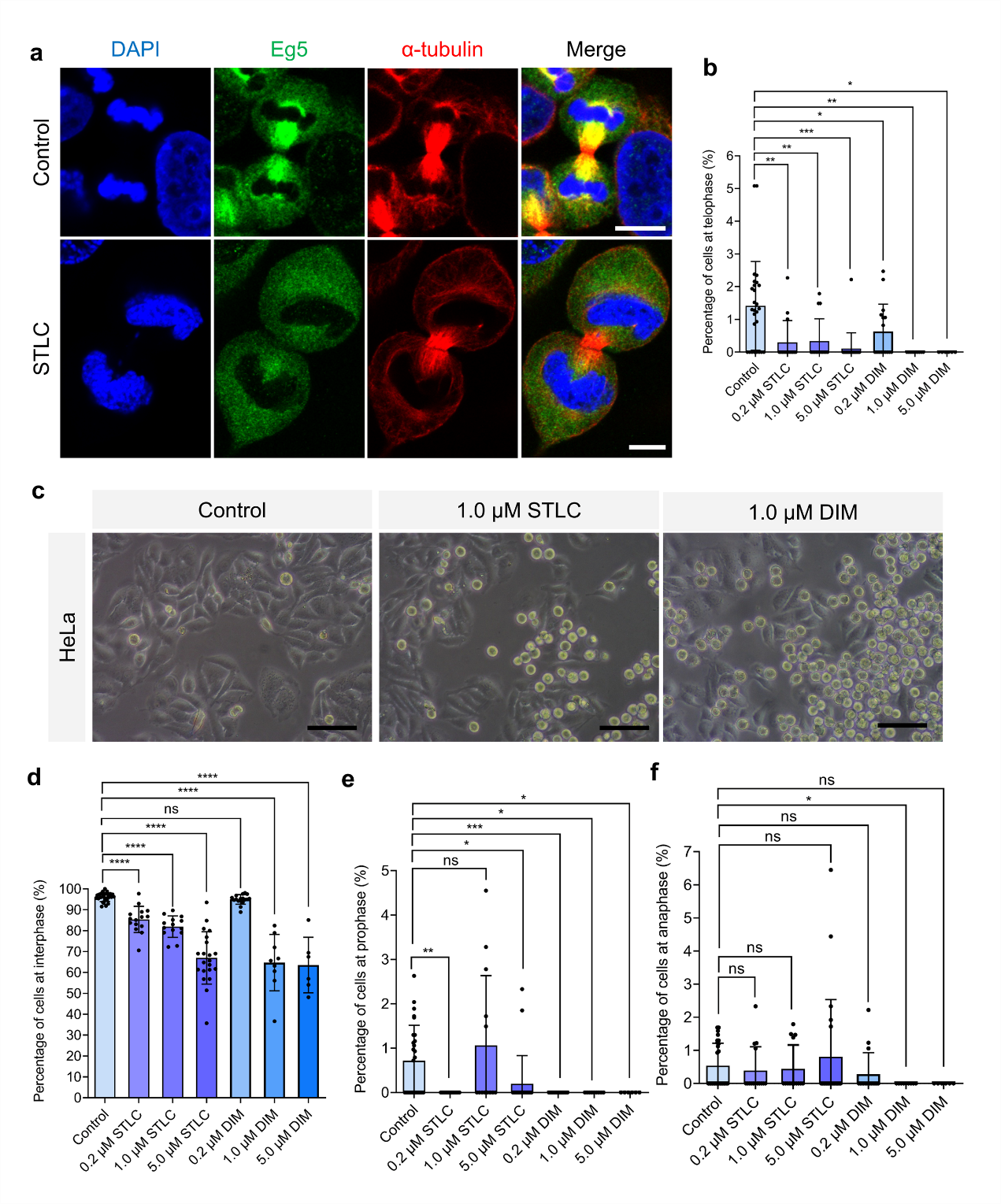
**

**Fig. S2** **Eg5 was required for chromosome segregation and stability during cell division.** **a** Representative immunofluorescence images of Eg5 and α-tubulin in HeLa cells during telophase. DAPI, blue; Eg5, green; α-tubulin, red. Scale bar, 10 μm. **b** The percentage of cells at telophase in the control, STLC, and Dimethylenastron groups. DIM indicates Dimethylenastron. **c** Representative image of HeLa cells in the Control, 1.0 μM STLC, and 1.0 μM Dimethylenastron groups. DIM indicates Dimethylenastron. Scale bar, 20 μm. **d** The percentage of cells at interphase in the control, STLC, and Dimethylenastron groups. **e** The percentage of cells at prophase in the control, STLC, and Dimethylenastron groups. **f** The percentage of cells at anaphase in the control, STLC, and Dimethylenastron groups. For all graphs, mean ± SEM was shown. Student’s *t*-test. ns, *p* > 0.05; *, *p* < 0.05; **, *p* < 0.01; ***, *p* < 0.001.

**Figure S3**

**
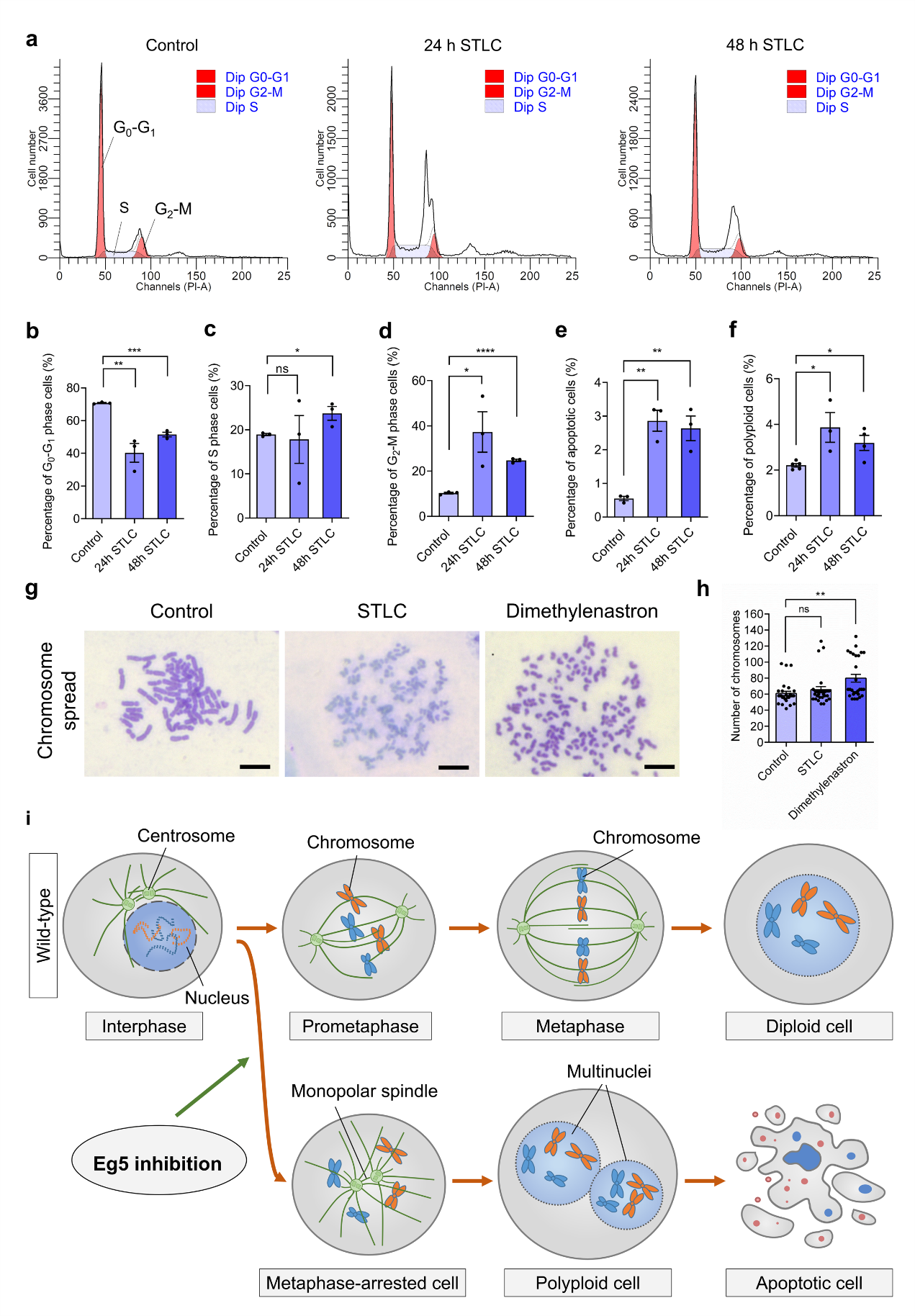
**

**Fig. S3** **Eg5 inhibition resulted in the G_2_-M phase arrest and the increase of polyploid cells. a** Flow cytometry analysis of HeLa cells in the control and STLC groups. HeLa cells were incubated with 1 μM STLC for 24 h and 48 h, respectively. N = 30000 cells were analyzed in each group. Group = 3. **b** The percentage of the G_0_-G_1_ phase cells in the control, 24 h STLC and 48 h STLC groups. Control, 60.36 ± 1.24%; 24 h STLC, 32.69 ± 6.10%; 48 h STLC, 45.20 ± 1.49%. **c** The percentage of the S phase cells in the control, 24 h STLC and 48 h STLC groups. Control, 18.44 ± 0.26%; 24 h STLC, 20.66 ± 2.49%; 48 h STLC, 19.89 ± 1.73%. **d** The percentage of the G_2_-M phase cells in the control, 24 h STLC and 48 h STLC groups. Control, 19.05 ± 0.54%; 24 h STLC, 36.29 ± 5.38%; 48 h STLC, 31.89 ± 0.83%. **e** The percentage of apoptotic cells in the control, 24 h STLC and 48 h STLC groups. Control, 0.55 ± 0.07%; 24 h STLC, 2.86 ± 0.31%; 48 h STLC, 2.64 ± 0.37%. **f** The percentage of polyploid cells in the control, 24 h STLC and 48 h STLC groups. Control, 3.54 ± 0.08%; 24 h STLC, 7.08 ± 0.54%; 48 h STLC, 7.14 ± 0.88%. **g** Karyotype analysis of HeLa cells in the control, STLC, and Dimethylenastron groups. Scale bar, 10 μm. **h** The number of chromosomes in HeLa cells in the control, STLC, and Dimethylenastron groups. Control, 60.93 ± 2.67; STLC, 65.6 ± 3.76; Dimethylenastron, 79.89 ± 4.84. N = 28. **i** Cellular mechanisms of Eg5 proteins in spindle assembly and chromosome alignment in HeLa cells during cell division. Eg5 inhibition results in the formation of the monopolar spindle, cell cycle arrest, and the increase of polyploid and apoptotic cells. For all graphs, mean ± SEM was shown. Student’s *t*-test. ns, *p* > 0.05; *, *p* < 0.05; **, *p* < 0.01; ***, *p* < 0.001.

**Figure S4**

**
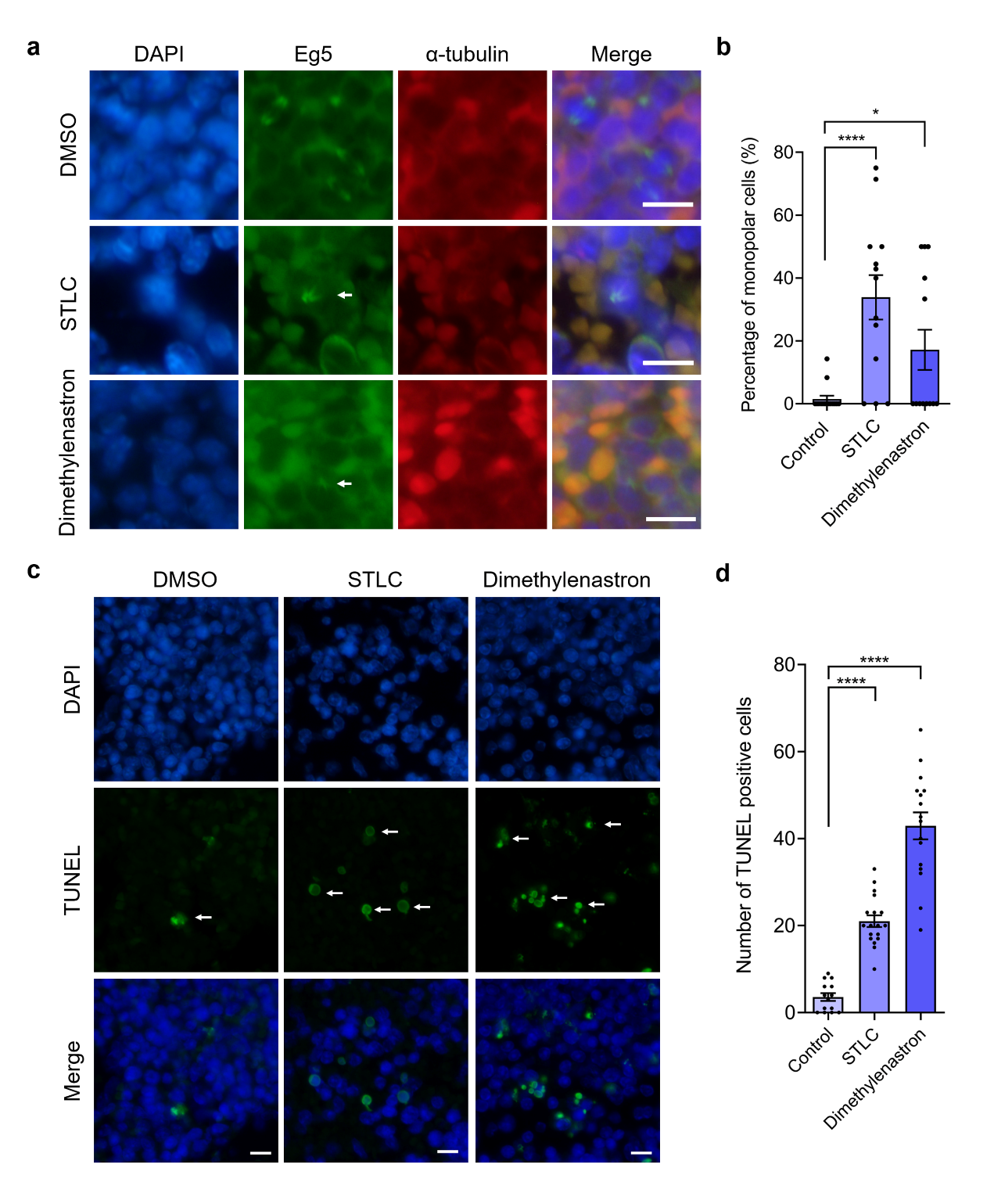
Fig. S4** Eg5 inhibition resulted in the monopolar spindle and apoptotic cells in the mouse spleen. **a** Representative immunofluorescence images of Eg5 and α-tubulin in mouse spleen cells in the control, STLC, and Dimethylenastron groups. DAPI, blue; Eg5, green; α-tubulin, red. The white arrow indicates the cell with monopolar spindle. Scale bar, 10 μm. **b** Percentage of monopolar cells in mouse spleen. DMSO, 1.51 ± 1.07, N = 15; STLC, 33.87 ± 7.07, N = 13; Dimethylenastron, 17.18 ± 6.39, N = 13. **c** Representative images of mouse spleen in the control, STLC, and Dimethylenastron groups. DAPI, blue; Eg5, green; α-tubulin, red. The white arrow indicates apoptotic cells. Scale bar, 10 μm. **d** Relative number of TUNEL positive cells in mouse spleen. DMSO, 3.57 ± 0.89, N = 14; STLC, 21.00 ± 1.35, N = 18; Dimethylenastron, 42.94 ± 3.09, N = 16. For all graphs, mean ± SEM was shown. Student’s *t*-test. ns, *p* > 0.05; *, *p* < 0.05; ****, *p* < 0.0001.

**Figure S5**

**
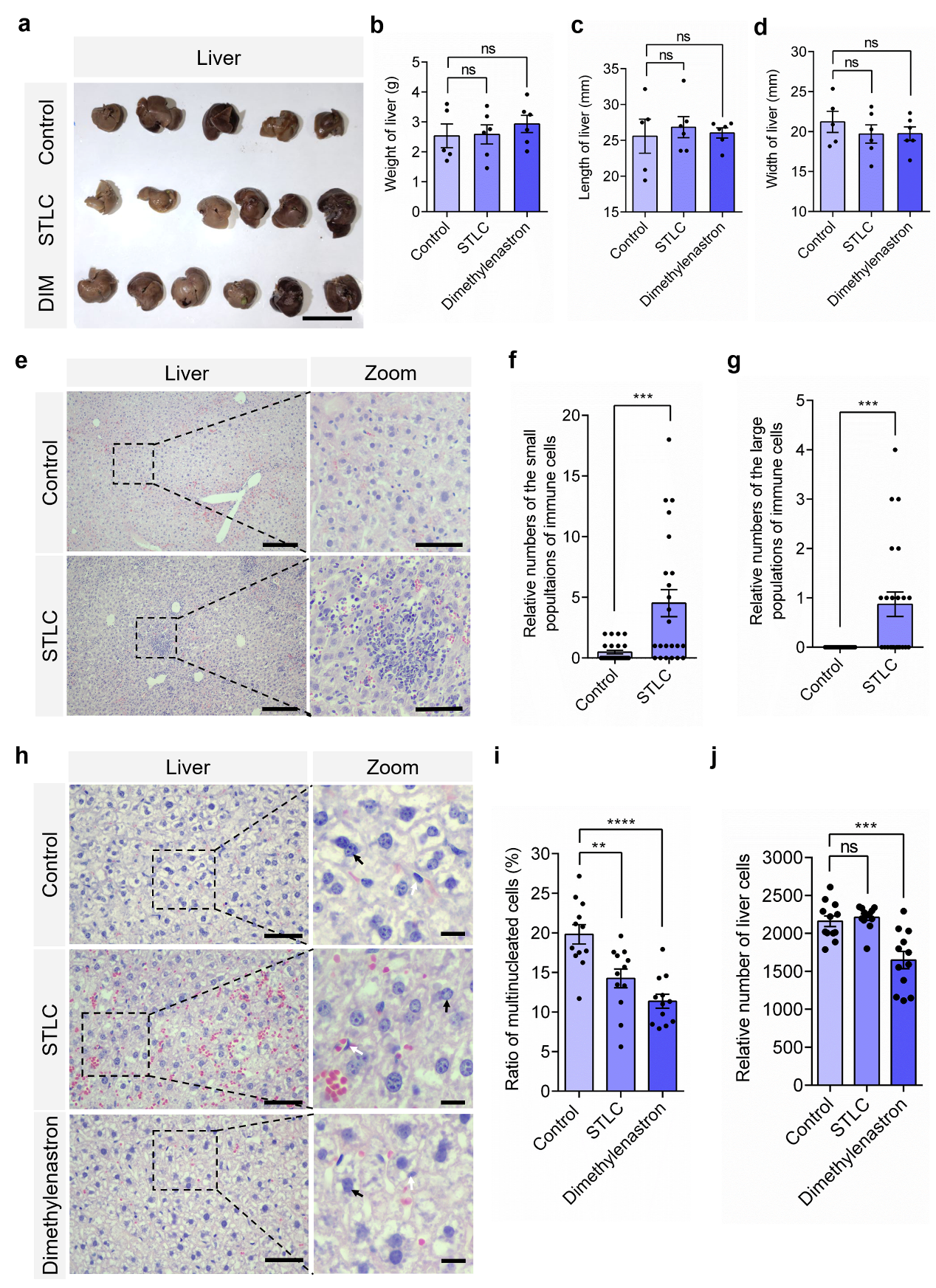
**

**Fig. S5** **Eg5 inhibition led to the increase of immune cells in mouse liver.** **a** Representative images of mouse liver in the control, STLC, and Dimethylenastron groups. Scale bar, 2 cm. **b** The weight of the livers in the control, STLC, and Dimethylenastron groups. Control, 2.53 ± 0.40 g, N = 5; STLC, 2.58 ± 0.32 g, N = 6; Dimethylenastron, 2.93 ± 0.29 g, N = 6. **c** The length of mouse livers. Control, 25.59 ± 2.37 mm, N = 5; STLC, 26.84 ± 1.48 mm, N = 6; Dimethylenastron, 26.03 ± 0.71 mm, N = 6. **d** The width of mouse livers. Control, 21.21 ± 1.33 mm, N = 5; STLC, 19.69 ± 1.13 mm, N = 6; DIM, 19.74 ± 0.86 mm, N = 6. **e** Representative HE images of mouse liver in the control and STLC groups. Scale bar, 200 μm. In the zoom, scale bar, 50 μm. **f** Relative numbers of the small population of immune cells in mouse livers of control and STLC groups. The number of immune cells in a small population is defined as cell number less than 20. Control, 0.48 ± 0.15; STLC, 4.52 ± 1.11. Control, group = 25; STLC, group = 23. **g** Relative numbers of the large population of immune cells in mouse liver of control and STLC groups. The number of immune cells in a large population is define as cell numbers more than 20. Control, 0.00 ± 0.00; STLC, 0.87 ± 0.25. Control, group = 25; STLC, group = 24. **h** Representative images of mouse livers in the control, STLC and Dimethylenastron groups. Scale bar, 50 μm. In the zoom, scale bar, 10 μm. **i** The percentage of multinucleated cells in the control, STLC, and Dimethylenastron groups. Control, 19.81 ± 1.22%, group = 12; STLC, 14.25 ± 1.19%, group = 12; Dimethylenastron, 11.36 ± 0.88%, group = 12. **j** Relative number of liver cells in the control, STLC, and Dimethylenastron groups. Control, 2160 ± 71, group = 12; STLC, 2213 ± 43, group = 12; Dimethylenastron, 1648 ± 112, group = 12. For all graphs, mean ± SEM was shown. Student’s *t*-test. ns, *p* > 0.05; *, *p* < 0.05; **, *p* < 0.01; ***, *p* < 0.001.

**Figure S6**

**
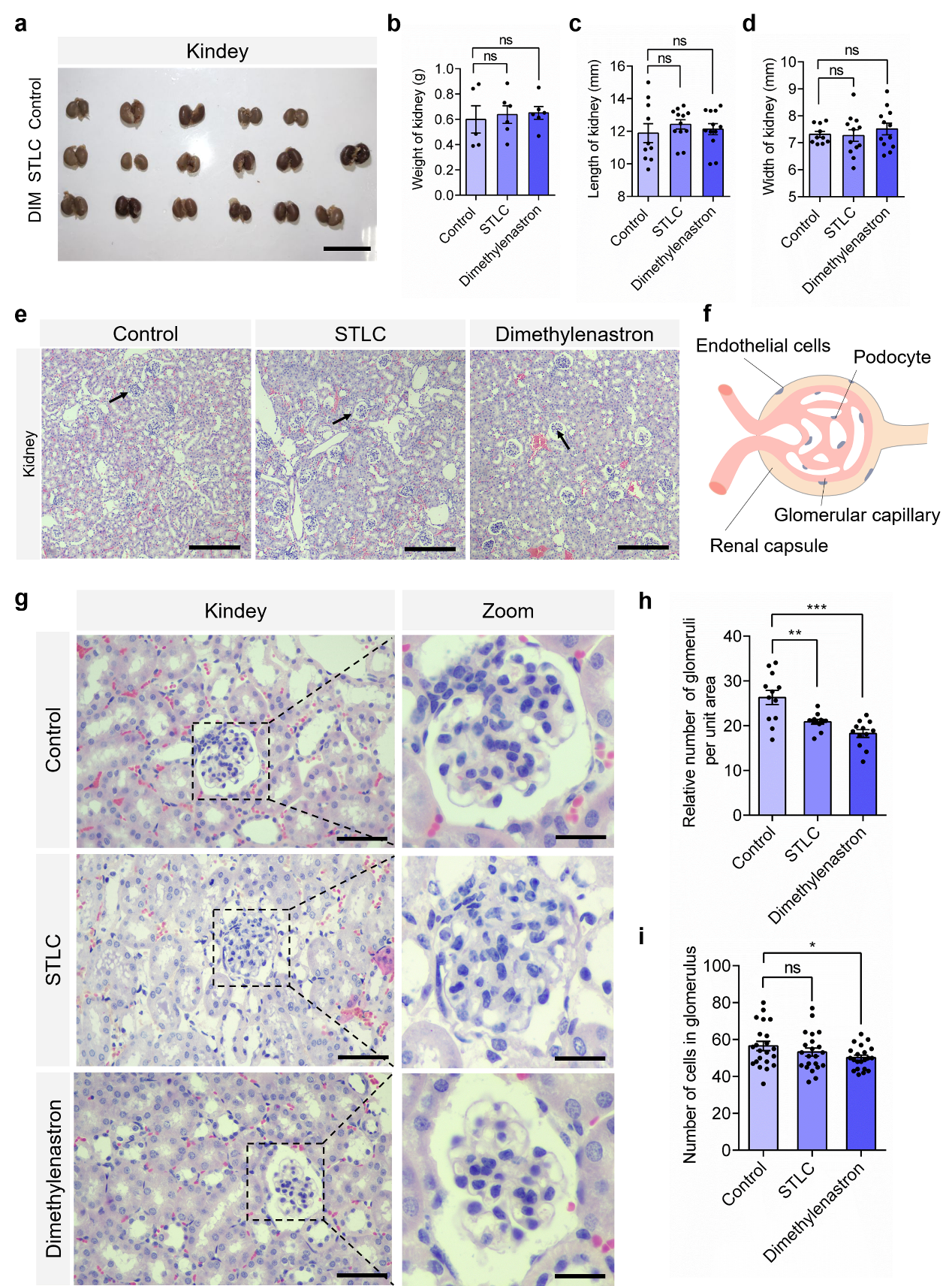
**

**Fig. S6 The effects of Eg5 inhibition on the development of mouse kidneys. a** Representative HE images of mouse kidneys in the control, STLC, and Dimethylenastron groups. Scale bar, 2 cm. **b** The weight of mouse kidneys in the Control, STLC, and Dimethylenastron groups. N = 6. **c** The length of mouse kidneys in the control, STLC, and Dimethylenastron groups. N = 6. **d** The width of mouse kidneys in the control, STLC, and Dimethylenastron groups. N = 6. **e** Representative HE images of mouse kidneys in the control, STLC, and Dimethylenastron groups. Arrows indicate renal corpuscles. Scale bar, 100 μm. **f** The model of mouse renal corpuscles in the kidney. **g** Representative HE images of the mouse glomeruli in the control, STLC, and Dimethylenastron groups. Scale bar, 50 μm. In the zoom, scale bar, 20 μm. **h** The relative number of glomeruli per unit area. Control, 26.29 ± 1.59; STLC, 20.88 ± 0.53. Dimethylenastron, 18.25 ± 0.87. **i** The number of cells in the mouse glomerulus. Control, 56.50 ± 2.47; STLC, 53.23 ± 2.20. Dimethylenastron, 50.19 ± 1.36. DMSO, N = 22, group = 6; STLC, N = 22, group = 6; Dimethylenastron, N = 21, group = 6. For all graphs, mean ± SEM was shown. Student’s *t*-test. ns, *p* > 0.05; *, *p* < 0.05; **, *p* < 0.01; ***, *p* < 0.001.

**Figure S7**

**
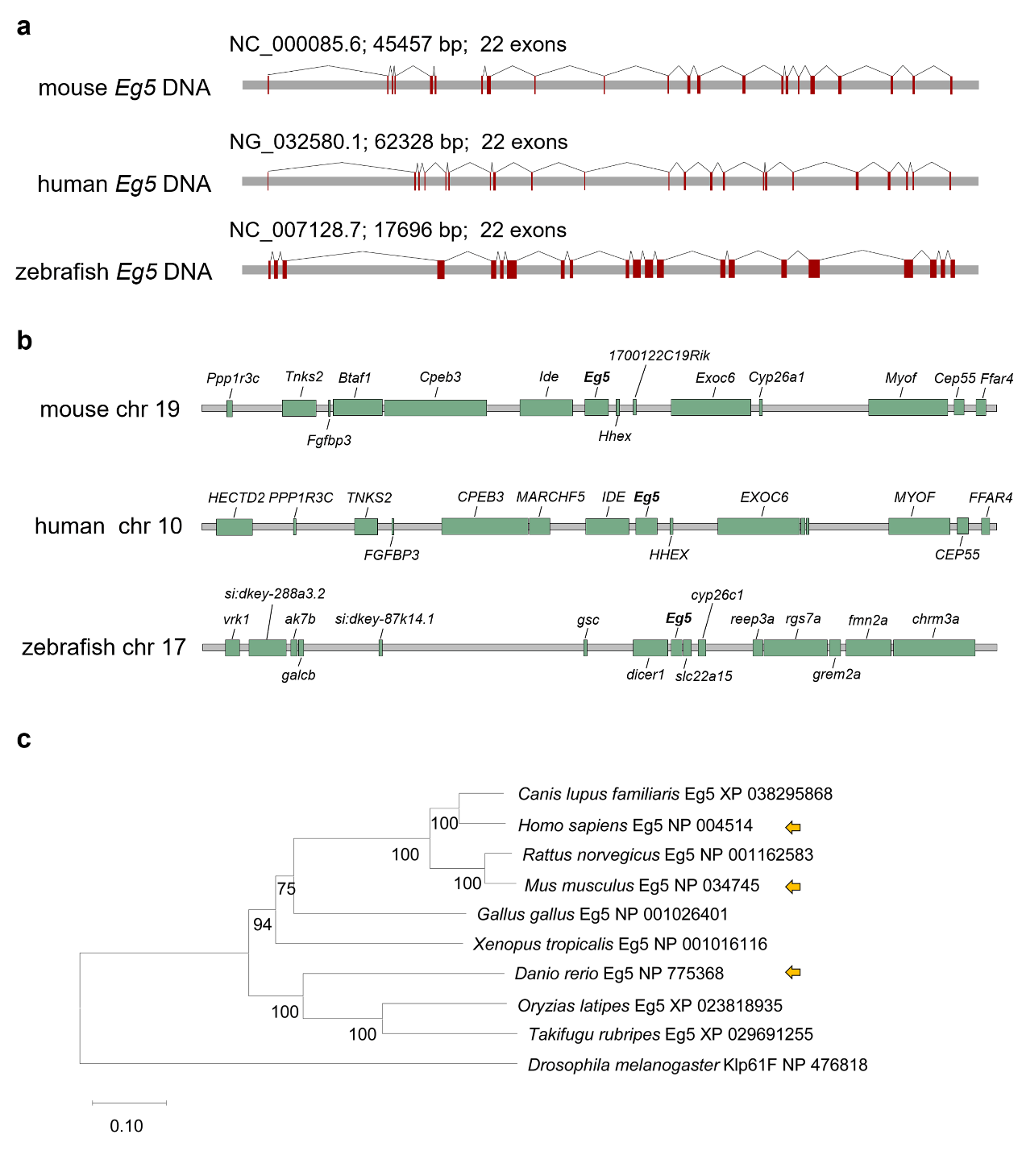
**

**Fig. S7** **The genetic and evolutionary analysis of mouse, human, and zebrafish kinesin-5 Eg5 genes and proteins.** **a** Representative images of the exons and introns of the mouse, human, and zebrafish *Eg5* genes. The GenBank accession number, gene length, and the number of exons were shown. The red box indicates the exons. The fold line and grey box indicate the introns. **b** The chromosomal regions of zebrafish chromosome 17 around the zebrafish *Eg5* gene are compared with the regions of human chromosome 10 and mouse chromosome 19. The adjacent genes of *Eg5* genes were shown. **c** Phylogenetic analysis of Eg5 protein sequences in multiple model organisms. The evolutionary tree was analyzed using the Neighbor-Joining method in the MEGA X software. The evolutionary distances were analyzed using the Poisson correction method. The percentage of replicate trees in which the associated taxa clustered together in the bootstrap test (1000 replicates) were indicated proximal to the branches. The organism names and the GenBank accession number of Eg5 proteins in diverse organisms were shown.

**Figure S8**

**
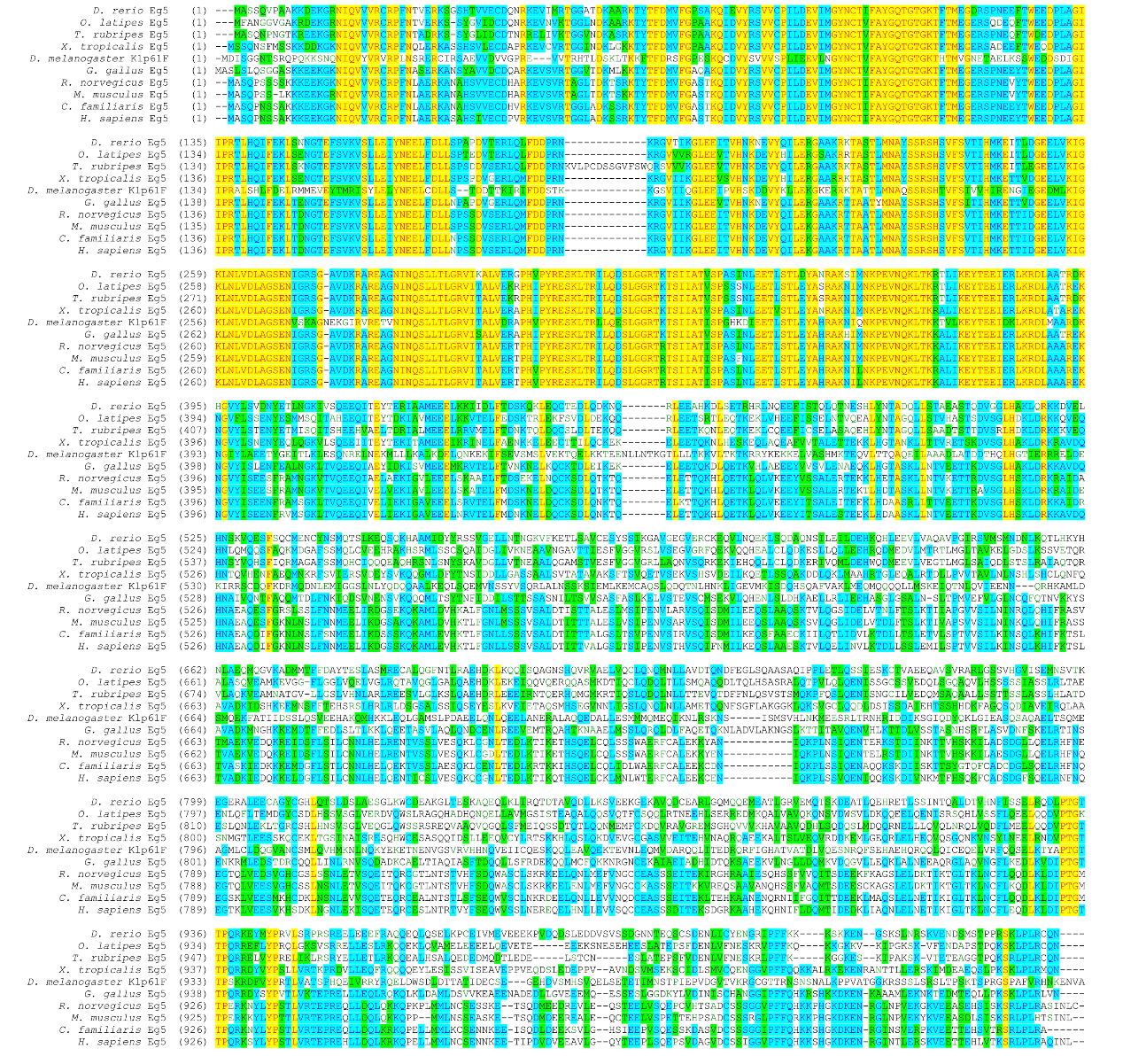
**

**Fig. S8** **Multiple sequence alignments of kinesin-5 Eg5 proteins in model organisms.** The amino acid sequence of Eg5 proteins in model organisms was aligned and analyzed using Align X of Vector NTI 11.5 software (Invitrogen). The GenBank accession numbers are listed as follows: *Danio rerio* Eg5 NP_775368.2, *Oryzias latipes* Eg5 XP_023818935.1, *Takifugu rubripes* Eg5 XP_029691255.1, *Xenopus tropicalis* Eg5 NP_001016116.2, *Drosophila melanogaster* Klp61F NP_476818.1, *Gallus gallus* Eg5 NP_001026401.1, *Rattus norvegicus* Eg5 NP_001162583.1, *Mus musculus* Eg5 NP_034745.1, *Canis lupus familiaris* KIF11 XP_038295868.1, *Homo sapiens* Eg5 NP_004514.2. The yellow regions indicate identical amino acids. The green regions indicate the highly conserved amino acids. The blue regions indicate the conserved amino acids. The white regions indicate the unconservative amino acids.

**Figure S9**

**
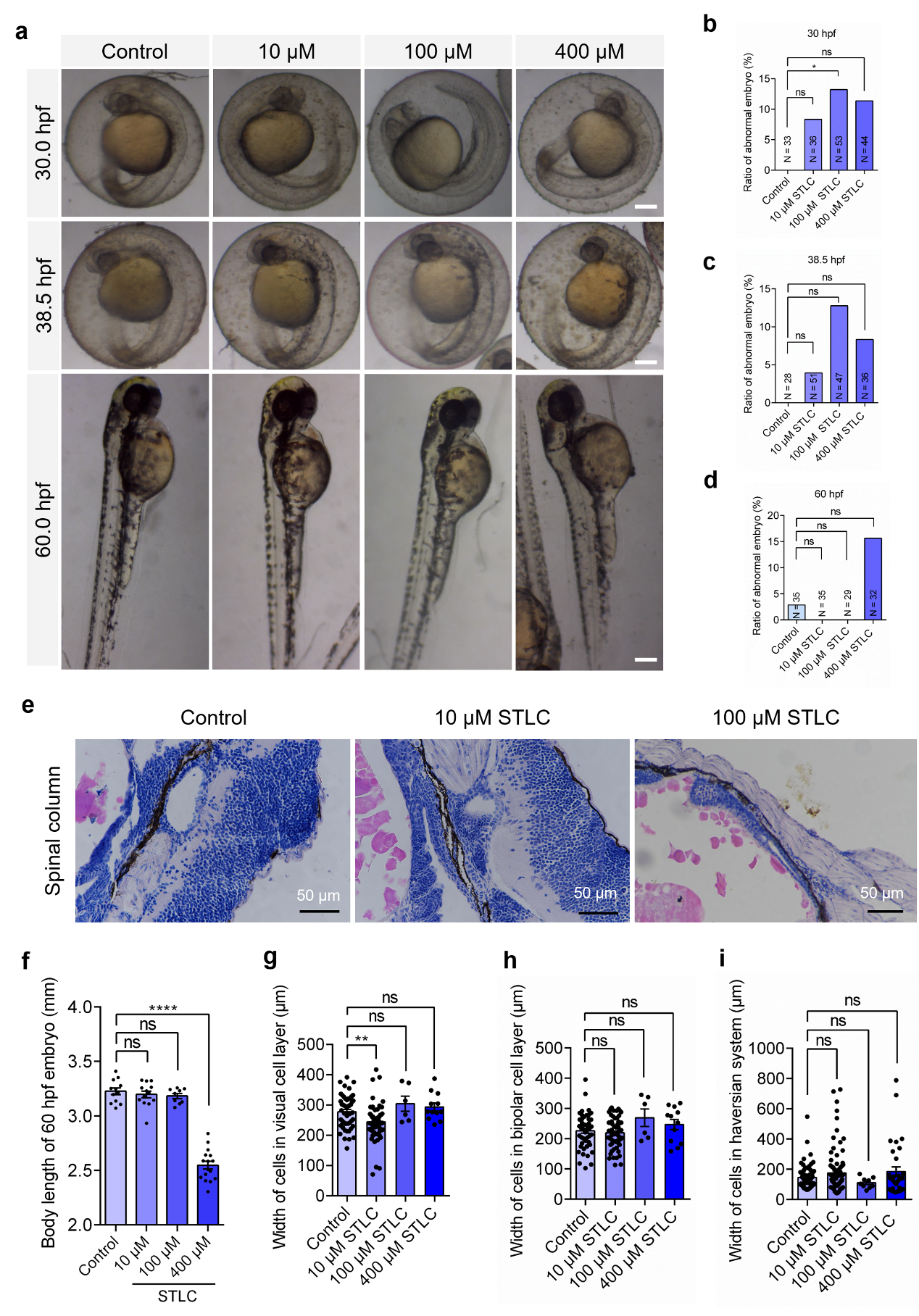
**

**Fig. S9** **Eg5 regulated the development of the somites in the zebrafish model.** **a** Representative images of the zebrafish embryos at 30.0, 38.5, and 60.0 hpf in the control and STLC groups. Scale bar, 200 μm. **b-d** The ratios of abnormal embryos at 30.0, 38.5, and 60.0 hpf in the control and STLC groups. In this graph, the chi-square test was used. N values were shown in the figure. ns, *p* > 0.05; *, *p* < 0.05. **e** Representative images of the zebrafish somites in the control and STLC groups. **f** The body length of 60 hpf zebrafish embryos in the control, 10 μM, 100 μM, and 400 μM STLC groups. **g** The width of cells in the visual cell layer in the zebrafish eyes of the control, 10 μM, 100 μM, and 400 μM STLC groups. **h** The width of cells in the bipolar cell layer in the zebrafish eye of the control, 10 μM, 100 μM, and 400 μM STLC groups. **i** The width of cells in the haversian system in the zebrafish eye of the control, 10 μM, 100 μM, and 400 μM STLC groups. Mean ± SEM was shown. Student’s *t*-test. ns, *p* > 0.05; **, *p* < 0.001; ***, *p* < 0.001.
