# Supplementary material for: Kinesin-5 Eg5 is essential for spindle assembly, chromosome stability and organogenesis in development
Source: Cell Death Discov. 2022 Dec 13;8:490. doi: 10.1038/s41420-022-01281-1 (PMC9747790; doi:10.1038/s41420-022-01281-1)
Supplement: Supplementary file 2 — Supplemental Table S1-S2 [file 41420_2022_1281_MOESM2_ESM.docx]

**Table S1. Percentages (%) of amino acid sequence identity for kinesin-5 Eg5 proteins in model organisms.**

|  | **zebrafish** | **medaka** | **torafugu** | **fruit fly** | **frog** | **chicken** | **mouse** | **rat** | **dog** | **human** |
| --- | --- | --- | --- | --- | --- | --- | --- | --- | --- | --- |
| **zebrafish** | 100 | 56.2 | 54.3 | 30.6 | 54.0 | 52.0 | 47.7 | 47.9 | 48.8 | 48.1 |
| **medaka** |  | 100 | 68.0 | 29.9 | 53.0 | 50.3 | 48.1 | 47.9 | 47.7 | 47.6 |
| **torafugu** |  |  | 100 | 29.4 | 49.5 | 48.1 | 47.2 | 46.5 | 46.8 | 46.6 |
| **fruit fly** |  |  |  | 100 | 31.6 | 31.6 | 31.4 | 31.2 | 32.2 | 31.5 |
| **frog** |  |  |  |  | 100 | 58.5 | 54.0 | 54.4 | 55.4 | 55.3 |
| **chicken** |  |  |  |  |  | 100 | 56.4 | 56.6 | 58.5 | 59.3 |
| **mouse** |  |  |  |  |  |  | 100 | 92.0 | 80.2 | 79.6 |
| **rat** |  |  |  |  |  |  |  | 100 | 80.9 | 80.2 |
| **dog** |  |  |  |  |  |  |  |  | 100 | 88.1 |
| **human** |  |  |  |  |  |  |  |  |  | 100 |

**Note:** zebrafish, *Danio rerio*; medaka (Japanese medaka), *Oryzias latipes*; torafugu, *Takifugu rubripes*; fruit fly, *Drosophila melanogaster*; frog (African clawed frog), *Xenopus laevis*; chicken, *Gallus gallus*; mouse (house mouse), *Mus musculus*; rat (Norway rat), *Rattus norvegicus*; dog, *Canis lupus familiaris*; human, *Homo sapiens*.

**Table S2. Percentages (%) of amino acid sequence identity for motor domain of kinesin-5 Eg5 in model organisms.**

|  | **zebrafish** | **medaka** | **torafugu** | **fruit fly** | **frog** | **chicken** | **mouse** | **rat** | **dog** | **human** |
| --- | --- | --- | --- | --- | --- | --- | --- | --- | --- | --- |
| **zebrafish** | 100 | 87.2 | 80.6 | 55.1 | 85.2 | 85.9 | 86.0 | 85.5 | 86.1 | 85.8 |
| **medaka** |  | 100 | 84.6 | 55.6 | 85.6 | 82.9 | 83.0 | 82.2 | 82.5 | 82.2 |
| **torafugu** |  |  | 100 | 49.5 | 79.0 | 77.9 | 78.4 | 78.4 | 78.4 | 78.4 |
| **fruit fly** |  |  |  | 100 | 56.3 | 54.6 | 55.6 | 55.9 | 56.2 | 55.6 |
| **frog** |  |  |  |  | 100 | 86.9 | 86.1 | 86.6 | 86.0 | 86.0 |
| **chicken** |  |  |  |  |  | 100 | 87.3 | 88.3 | 88.6 | 88.1 |
| **mouse** |  |  |  |  |  |  | 100 | 98.3 | 96.4 | 95.5 |
| **rat** |  |  |  |  |  |  |  | 100 | 97.5 | 96.2 |
| **dog** |  |  |  |  |  |  |  |  | 100 | 99.2 |
| **human** |  |  |  |  |  |  |  |  |  | 100 |

**Note:** zebrafish, *Danio rerio*; medaka (Japanese medaka), *Oryzias latipes*; torafugu, *Takifugu rubripes*; fruit fly, *Drosophila melanogaster*; frog (African clawed frog), *Xenopus laevis*; chicken, *Gallus gallus*; mouse (house mouse), *Mus musculus*; rat (Norway rat), *Rattus norvegicus*; dog, *Canis lupus familiaris*; human, *Homo sapiens*.
